# Dry Development of Dry Coated Sn‐Based Inorganic Resist for Defect‐Suppressed and High‐Resolution Patterning Process

**DOI:** 10.1002/smtd.70794

**Published:** 2026-06-23

**Authors:** Hee Ju Kim, Min Cheol Kim, Geun Young Yeom

**Affiliations:** ^1^ School of Advanced Materials Science and Engineering Sungkyunkwan University Suwon South Korea; ^2^ Department of Display Engineering Sungkyunkwan University Suwon South Korea; ^3^ SKKU Advanced Institute of Nano Technology (SAINT) Sungkyunkwan University Suwon South Korea

**Keywords:** dry development, dry resist, inorganic photoresist, metal oxide resist, plasma development, pulsed plasma

## Abstract

The advancement of semiconductor technology has driven the need for next‐generation patterning techniques that ensure high fidelity and sub‐10 nm critical dimension (CD) control. In this study, we propose a novel plasma development process using an asynchronously pulsed plasma for the development of Sn‐based inorganic dry resists. The asynchronously pulsed plasma, especially characterized by the alternate application of source and bias RF power, enables high chemical contrast while minimizing physical damage through precise control of radical chemisorption and energy/flux of reactive ions. Compared to wet development, this pulsed plasma development technique substantially suppresses defectivity, including line bridging and scum formation, while reducing the line‐edge roughness (LER) by approximately 29%. This improvement enables higher patterning fidelity for sub‐10 nm features and expands the lithography process margin. In addition, because positively charged ions in the plasma remove the resist through combined chemical and physical pathways, the process mitigates defect mechanisms associated with airborne molecular contamination (AMC) and resist aging. Consequently, overall process stability is enhanced, and the operational process window is broadened. These results demonstrate the potential of pulsed plasma development as a highly selective, low‐damage, and defect‐suppressing strategy for future high‐resolution lithographic applications regardless of whether it is spin‐coated or dry deposited.

## Graphical abstract

This work compares conventional wet development and asynchronously pulsed plasma development for Sn‐based inorganic resists under delay and AMC exposure. While wet development induces CD variation, scumming, and bridging, pulsed plasma enables selective removal of non‐exposed regions, mitigating contamination effects and achieving defect‐suppressed, high‐fidelity nanoscale patterning.

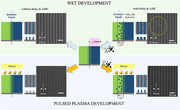

## 1 Introduction

Since extreme ultraviolet lithography (EUVL) was proposed as a new technology for semiconductor nanoscale patterning in the late 1980s [1, 2], there have been many studies and developments, and it is currently being widely used in high‐volume manufacturing [3] of semiconductor devices. The capability of EUVL, significantly reducing the process complexity compared to ArF immersion multiple patterning techniques, has been demonstrated in terms of its productivity [4, 5, 6, 7], resulting from its short wavelength of the light source. Despite these advantages, EUVL still faces several critical challenges [8, 9, 10], including limited source power, mask contamination, and narrow depth of focus, etc.

Among these limitations, the EUV photoresist remained as a primary bottleneck. Chemically amplified resists (CARs), the standard lithography materials for conventional photolithography, exhibit performance degradation under EUV conditions due to their intrinsic stochastic behavior. Especially, the random distribution of photoacid generators and quenches resulted in stochastic defects [11, 12] such as bridging and line breaks. In addition, CARs, having large molecular structures, suffer from relatively high line edge roughness (LER) and line width roughness (LWR). Also, as the feature size is decreased, low resistance of the CARs [13] is one of the problems during the etching process for transferring the patterns to the underlayer, because the defects could be transferred to the final feature, causing degradation of devices.

For these reasons, inorganic photoresists, particularly metal oxide resists, have been suggested as promising alternatives [14, 15, 16, 17, 18, 19, 20]. Metal oxide resists offer low molecular weight, improved EUV absorption cross‐section, and enhanced etch resistance, leading to better fidelity. Commercially available metal oxide resists have demonstrated sub‐10 nm patterning fidelity, developed by Inpria and Lam Research, and also evaluated for high numerical EUV platforms, too. However, even with the metal oxide systems, the conventional development process still relies on solvents. Wet development could induce pattern collapse due to the capillary force during drying after the development, and it could be critical for narrower features. To overcome these problems, dry development techniques have been reported. Macdonald et al. [21, 22] reported an O_2_ plasma development method for a CAR‐type photoresist. Hydrogensilsesquioxane (HSQ), which has been used for e‐beam photoresist, was also developed by a Cl_2_ plasma [23]. TEL Corp [24, 25] also reported the dry development rinse process (DDRP)/organic dry development rinse process (ODDR), skipping drying up the developer, and by applying DDR materials followed by an O_2_ plasma treatment. LAM research reported a dry patterning process that uses both the chemical vapor deposition of Sn oxide‐based photoresist, and the dry development by vapor‐phase removal and He plasma etching [26, 27]. Also, Corkery et al. [28] and Seok et al. [29] reported a gas‐phase development using HfacH.

Although previous studies have demonstrated alternative development approaches capable of mitigating defects associated with solvent‐based processes, the risk of residual contamination remains. For example, a patent by Lam Research [26, 27] reports that gas‐phase etching using HBr or HCl for the removal of unexposed regions can leave residues behind; therefore, it proposes a subsequent He plasma sputtering step to eliminate them. In addition, degradation of patterning performance due to airborne molecular contamination (AMC) and post‐exposure delay (PED) [30, 31, 32] remains a significant challenge, as both factors can induce variation in critical dimension (CD) and alter the resist sensitivity.

In this study, we propose an asynchronously pulsed plasma development process for patterning metal‐oxide resists. By decoupling the source and bias RF power, the plasma process enables independent control of radical and ion fluxes [33, 34], allowing the high‐selective and low‐damage removal of non‐exposed regions. The effects of key process parameters such as the duty ratio of the pulse (source and bias) on development characteristics are investigated. Etching behaviors of both non‐exposed and exposed regions are compared, and performance is evaluated against conventional wet development methods. This study provides the fundamental insights into a novel plasma development strategy offering enhanced defect suppression and process robustness for next‐generation lithography.

## 2 Experimental Details

**FIGURE 1:**
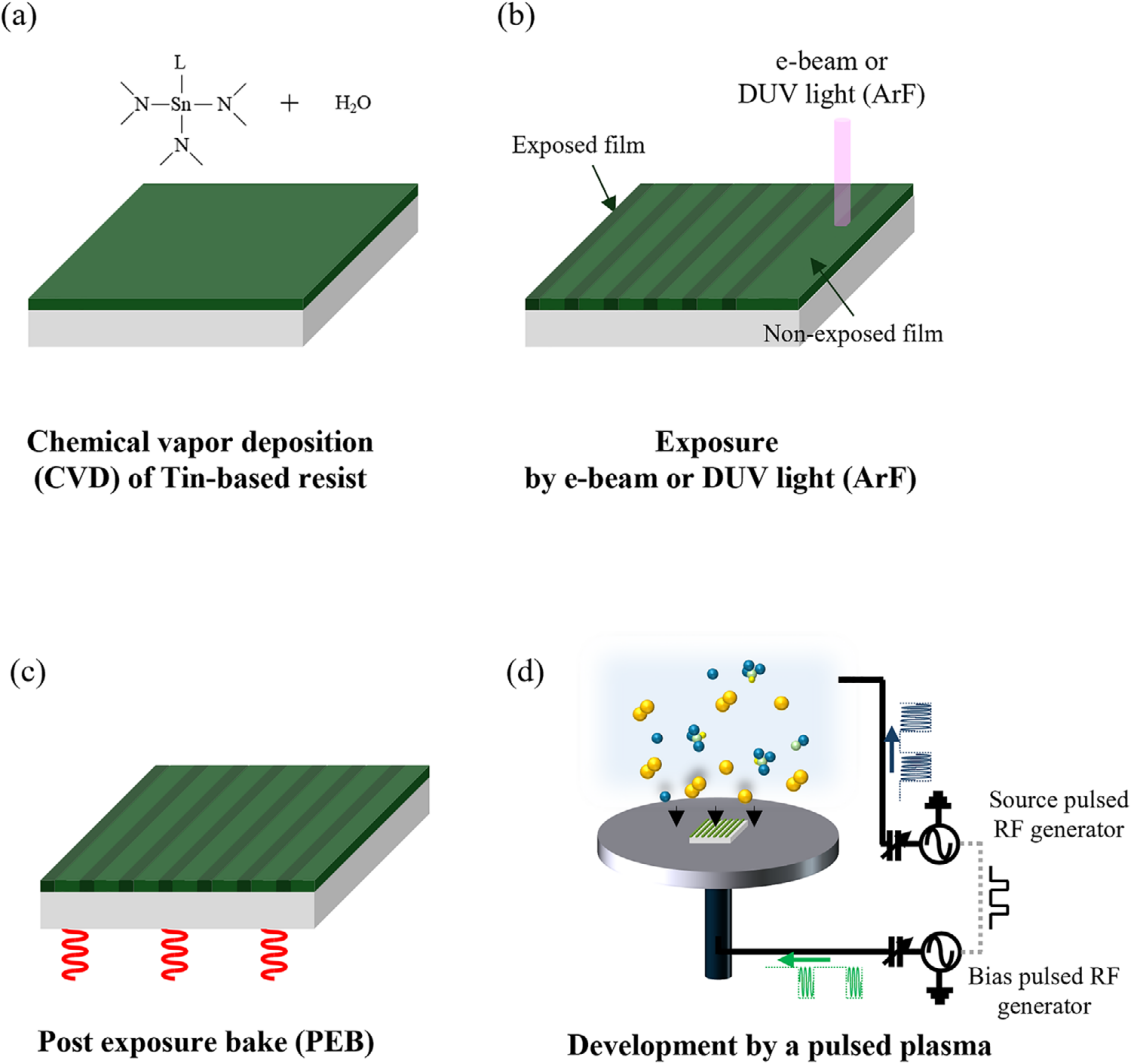
Schematic illustration of the overall process of plasma development for tin‐based dry resist. (a) Chemical deposition of Sn‐based resist, (b) exposure by an e‐beam or ArF light source, (c) post exposure bake, and (d) development by a pulsed plasma.

Figure 1 shows the schematic illustration of the plasma development process of Sn‐based dry resist. First, the resist film was deposited via chemical vapor deposition (CVD) using tert‐butyltris(dimetylamino)tin (t‐BuSn(NMe_2_)_3_) as the precursor, with Ar as a carrier gas and water vapor as a reactant. The dry resist CVD process was conducted at 70°C and 2 Torr [35]. The film thickness was controlled to approximately 38 nm (See Figures S1 and S2 for the analysis of the deposited films by scanning electron microscopy (SEM) and X‐ray photoelectron spectroscopy (XPS)). After the deposition, the films were patterned using either deep ultraviolet (DUV) lithography and electron‐beam lithography. For DUV lithography, an ArF dry scanner (ASML, XT1250D, 193 nm, dose 50 mJ/cm^2^) was used to create 500 nm line patterns. These DUV‐exposed samples were used to measure the etch rate (ER) of the resist and development selectivity from the ratio of non‐exposed resist ER to exposed resist ER. For the e‐beam lithography process (RAITH, EBPG5150 Plus), samples were exposed to e‐beams at doses ranging from 1000 to 2000 µC/cm^2^ with an acceleration voltage of 100 kV and a beam size of 10 nm. After exposure, post‐exposure bake (PEB) was performed at 250°C for 5 min on a hot plate in ambient air.

The asynchronously pulsed plasma development process was conducted in a 200 mm diameter inductively coupled plasma (ICP) etch system (modified STS‐PLC). 13.56 MHz radio frequency (RF) power (SEREN, R10001) was supplied to the source antenna through a matching network. A pulse signal was extracted from the source generator to trigger the bias RF power generating system, which consisted of a digital delay generator (DDG; SRS, DG645), a signal generator (HP, 8657B) set at 12.56 MHz, and an RF amplifier (ENI, A1000). Pulse repetition frequency (PRF) was kept at 500 Hz, and the duty ratio (DR) of the source and bias pulses was varied from 0% to 75%. During the development of Sn‐based resist, a gas mixture of Cl_2_/CHF_3_/H_2_ (47/10/3 sccm) was used, with a chamber pressure of 100 mTorr. The substrate temperature was maintained at 0°C using a chiller.

Pattern profiles and residual thickness of the resist were characterized using field emission SEM (FE‐SEM; Hitach, S4700). For cross‐sectional imaging, the SEM was operated at 15 kV with 20 µA beam current, and samples were coated by Pt. The top view imaging was conducted at 7 kV with 10∼15 µA beam current. XPS (VG Microtech Inc., ESCA2000) was used to investigate the chemical composition of the resist surface. For the surface analysis, resist samples were exposed to the DUV light without any pattern.

## 3 Results and Discussion

As shown in Figure S2, XPS results clearly demonstrate that light exposure induces significant chemical changes in the resist, leading to a compositional contrast between exposed and non‐exposed regions. Especially, the exposed regions exhibit pronounced enrichment of metal‐oxide components, whereas the non‐exposed regions retain higher carbon contents [26, 27, 35]. This light‐induced chemical contrast forms the basis of a negative tone development strategy, wherein the carbon‐rich non‐exposed regions can be selectively removed by plasma, while the oxygen‐rich, metal‐oxide network in the exposed regions is preserved.

**FIGURE 2:**
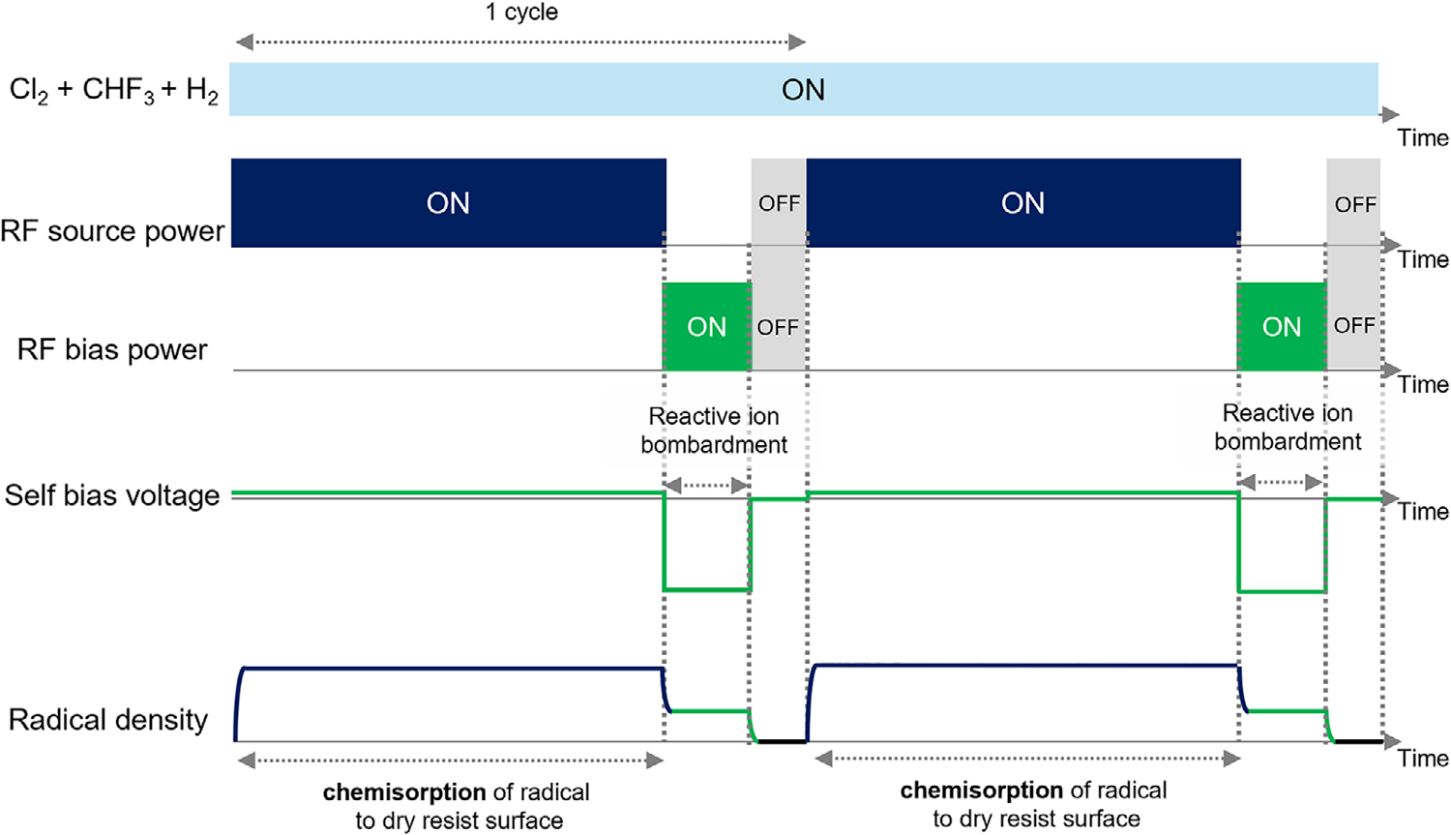
Concept of a pulsed plasma development process for a Sn‐based dry resist.

To achieve this goal, a pulsed plasma process was employed which decouples radical generation and ion bombardment by independently controlling the source and bias RF powers [33, 34, 36]. As illustrated in Figure 2, a gas mixture of Cl_2_, CHF_3_, and H_2_ is continuously supplied into the chamber. The source RF power is periodically turned on and off to control radical generation. At the end of each source pulse, the bias RF power was applied to induce directional reactive ion bombardment. During the source‐on period, high‐density inductively coupled plasma dissociates the feed gas, generating the highly reactive radical species that can chemisorb onto the resist surface. When the bias RF is turned on, a self‐bias voltage is generated, which accelerates positively charged ions toward the substrate. These ions selectively remove the less crosslinked, carbon rich regions through reactive ion etching, while minimizing loss of exposed regions. This cyclic plasma application is repeated until all non‐exposed regions are removed, ultimately resulting in the negative tone development.

**FIGURE 3:**
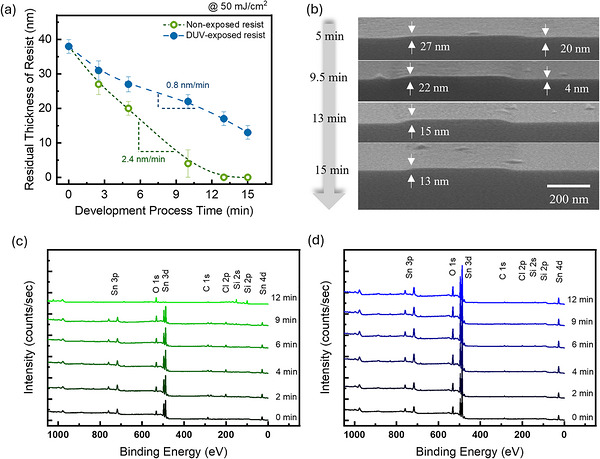
(a) Thickness change of 500 nm line patterns as a function of plasma development process time under a pulsed plasma condition. (b) Cross‐sectional SEM images of 500 nm line and space patterns developed under a pulsed plasma condition at various process times. XPS wide scan spectra of (c) non‐exposed and (d) exposed dry resist at different development times (Process conditions: RF power 500 W, bias voltage – 120 V_max_, source power duty ratio 75%, bias power duty ratio 4%, Cl_2_/CHF_3_/H_2_ (47/10/3 sccm), and operating pressure 100 mTorr).

To evaluate the development behavior of the dry resist, residual thickness and chemical composition changes were measured as a function of process time. Process conditions were RF power 500 W, bias voltage −40 V_max_, source power duty ratio 35%, bias power duty ratio 7.5%, Cl_2_/CHF_3_/H_2_ (47/10/3 sccm), and operating pressure 100 mTorr. (in Figure S3, the reason for the use of a gas mixture Cl_2_+CHF_3_+H_2_ instead of Cl_2_ only or Cl_2_+CHF_3_ is shown, and in Figure S4, the reason for the use of a low bias voltage for the optimized process is shown) As shown in Figure 3a, the thickness of the non‐exposed resist decreased linearly from ∼38 nm and was completely removed after 13 min, corresponding to an etch rate of ∼2.4 nm/min. In contrast, the exposed resist was etched at a slower rate (∼0.8 nm/min), with ∼15 nm remaining after 13 min, indicating selective removal of non‐exposed regions. For comparison, plasma development behavior under a continuous wave (CW) plasma (the same RF source and bias powers without pulsing) instead of the pulsed plasma is shown in Figure S5, and only 2 nm of exposed film remained after the development, resulting in poor selectivity of approximately 1.05.

Figure 3b shows the cross‐sectional SEM images obtained at various development times. With increasing process time, the non‐exposed regions were gradually removed, while the exposed regions remained intact. After 13 min of processing, the non‐exposed region was completely removed. Even under over etching condition (15 min processing), the exposed patterns were preserved without etching of the underlayer (25 nm of SiON).

Figure 3c,d show the XPS wide scan spectra of resist surfaces acquired at different development times. In the case of non‐exposed resist (Figure 3c), the Sn 3d and Sn 3p peaks disappeared completely after 12 min of plasma processing, while substrate‐related peaks (Si 2p and Si 2s) emerged. (The evolution of atomic percentages for both non‐exposed and exposed resist as a function of pulsed plasma development time is provided in Figure S6). These results indicate the complete removal of the non‐exposed resist layer and reveal the underlying layer (In this case, the Si substrate). In contrast, the exposed resist maintained a chemical composition similar to its initial state even after 12 min of development, consistent with Figure 3a,b. These results confirm successful negative‐tone development via selective removal of the carbon‐rich non‐exposed resist while preserving exposed regions.

**FIGURE 4:**
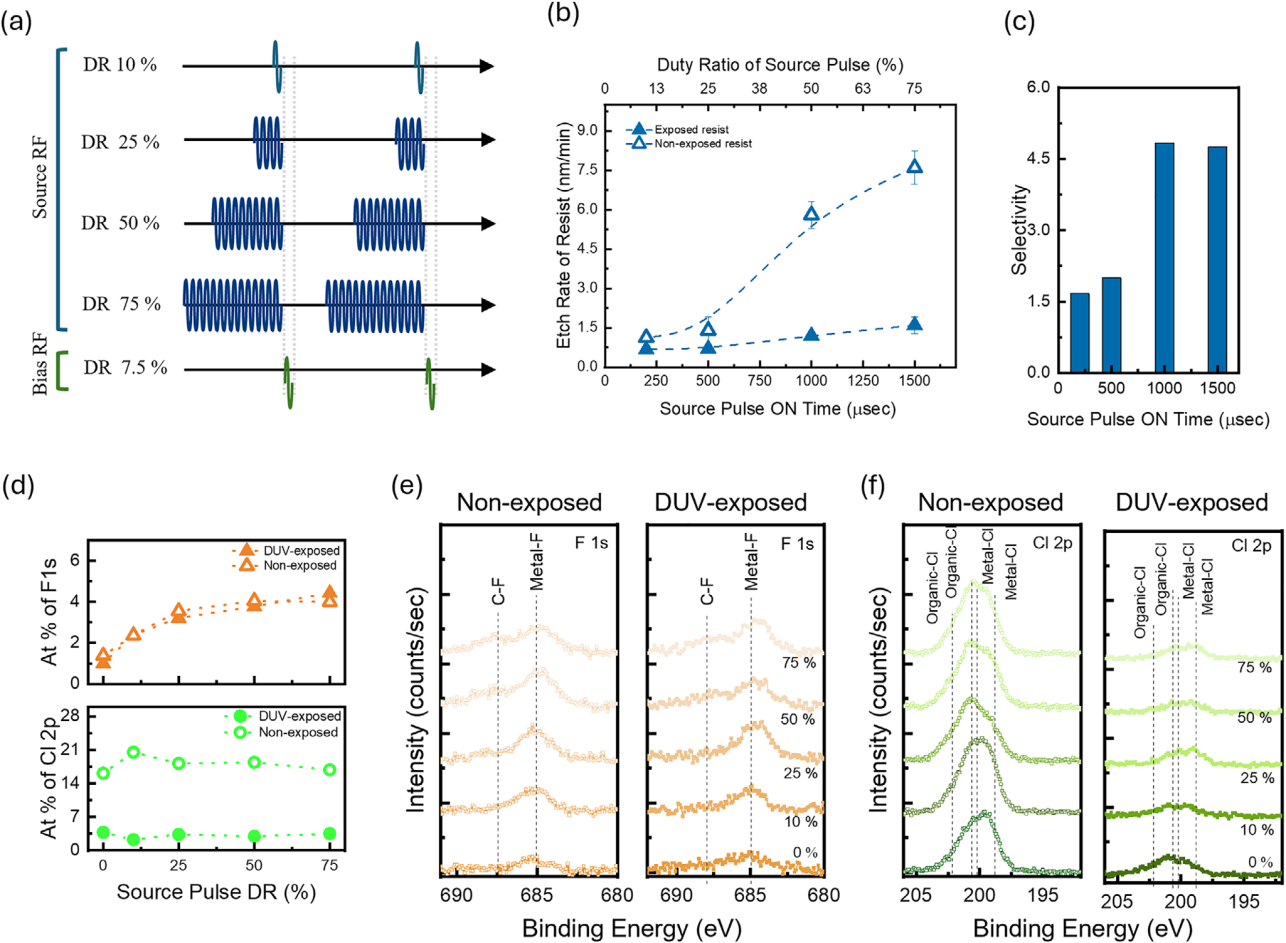
(a) Schematic illustration of different source pulse duty ratio (10%–75%), (b) etch rate of exposed and non‐exposed dry resist as a function of source pulse duty ratio (1000 µs = 50% duty ratio), (c) development selectivity as a function of source pulse duty ratio, and (d) atomic percentage changes of F 1s and Cl 2p after 2 min of process time at various source pulse duty ratios and its narrow scan spectra of (e) F 1s and (f) Cl 2p.

**FIGURE 5:**
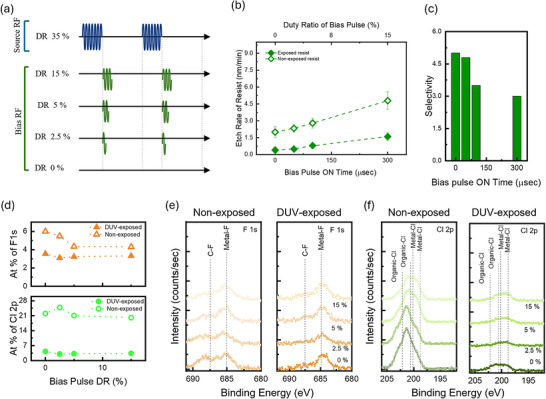
(a) Schematic illustration of different bias pulse duty ratio (%), (b) etch rate of exposed and non‐exposed dry resist as a function of bias pulse duty ratio (1000 µs = 50% duty ratio), (c) development selectivity as a function of bias pulse duty ratio, (d) atomic percentage changes of F 1s and Cl 2p after 4 min of process time at various bias pulse duty ratios and its narrow scan spectra of (e) F 1s and (f) Cl 2p.

To understand the influence of pulsed plasma parameters on development selectivity, the duty ratios of source and bias pulses were independently varied (Figure 4 for the source pulse, and Figure 5 for the bias pulse). Figure 4a illustrates the modulation of the source pulse duty ratio (%), while maintaining a fixed pulse repetition frequency (PRF) of 500 Hz and a constant bias pulse duty ratio of 7.5% (150 µsec). As shown in Figure 4b, the etch rate of the non‐exposed resist increased significantly with longer source pulse durations, from 1.1 nm/min at 10% DR to 7.6 nm/min at 75% DR, while the etch rate of exposed resist remained relatively unchanged (∼0.7 to 1.65 nm/min). The maximum selectivity of approximately 4.8 was observed at a source duty ratio of 50% (1000 µsec), as summarized in Figure 4c.

To understand the origin of the difference in ER between films and increased development selectivity with source pulse DR, surface chemical composition was analyzed by XPS after 2 min of process time at various source pulse duty ratios. As shown in Figure 4d, the atomic percentage of F increases with higher source DR for both exposed (from ∼1% at 0% of DR to ∼4.4% at 75% of DR) and non‐exposed (1.4% at 0% of DR to 4% at 75% of DR) samples. These results indicate enhanced adsorption of fluorine‐based species on both resist surfaces with increasing source pulse duration. However, a clear difference is observed in Cl behavior: Non‐exposed films exhibited a significantly higher Cl content, ranging from ∼16.1% to ∼20.5% at the initial DR of 10%. In Cl_2_ plasma etching, Sn─C bonds are primarily removed through the formation of volatile by‐products such as SnCl_4_ and SnCl_x_Hᵧ (x + y = 4). However, a short duty ratio limits the duration of plasma ignition, resulting in insufficient dissociation of Cl_2_ into reactive ions and radicals. Consequently, at short DRs, the continuous supply of Cl etchants is inadequate to fully remove unexposed Sn as volatile species, leading to the accumulation of partially chlorinated SnCl_x_ (x < 4) on the surface. As the DR increases, sustained Cl radical flux enables more efficient Sn removal, accompanied by an increase in the etch rate (ER). As a result, the Cl atomic% measured by XPS shows a slight decrease to ∼17%. Meanwhile, exposed films showed much lower Cl, remaining below 4% (∼3.8% to ∼2.3%) across all conditions. These results confirm that the carbon‐rich, loosely crosslinked non‐exposed films readily form C─Cl and Sn─Cl bonds with plasma‐generated Cl radicals, whereas the oxygen‐rich, dense Sn─O network of the exposed films exhibits minimal Cl adsorption [37].

Narrow scan spectra in Figure 4e (F 1s) and 4 (f) (Cl 2p) provide further insight into the chemical states. In the F 1s spectra (Figure 4e), peaks corresponding to metal‐F bonding (Sn─F, ∼685 eV) [38, 39] and C─F related peaks (688 eV) [38, 39] increase with increasing source DR, resulting in the formation of the fluorocarbon species on both resist surfaces. In contrast, the Cl 2p spectra shown in Figure 4f exhibit significant peak differences between non‐exposed and exposed resist, indicating the distinct chemical reactivity of each resist toward plasma‐generated reactive species. With the increase of source DR, higher organic‐Cl (202.2 and 200.6 eV) and higher metal‐Cl binding (198.8 and 200.2 eV) [37] were clearly observed in the non‐exposed resist. Also, Figure S7 shows the ratio of the organic‐Cl to metal‐Cl peak areas in Figure 4f of the non‐exposed resist as a function of source pulse DR. This trend indicates that more organic‐Cl binding formed than metal‐Cl as a function of source pulse DR, resulting in more formation of carbon‐chlorine binding on the non‐exposed resist surface due to longer high‐density plasma application time. (See Figure S8, showing the optical emission spectroscopy (OES) spectra of plasma as a function of DR of the source pulse. Cl‐related peaks gradually increased as a function of the source pulse DR, which originated from the plasma.

These findings demonstrate that the source duty ratio effectively modulates the surface chemistry of the resist by controlling radical density and adsorption time. The higher etch rate of the non‐exposed region originates from its increased reactivity toward radicals, which form volatile byproducts (SnCl_x_, SnH_x_, CF_x_, CCl_x_, etc.). In contrast, the exposed region, due to its dense Sn─O structure, exhibits greater resistance to chemical modification.

In contrast, the bias pulse duty ratio primarily influences reactive ion bombardment. Figure 5a illustrates the variation of bias pulse DR from 0% (0 µsec) to 15% (300 µsec), while the source pulse DR was kept at 35% (700 µsec). The etch rates of exposed (green filled rhombus) and non‐exposed resists (green empty rhombus) as a function of bias pulse DR are shown in Figure 5b. As the bias pulse duty ratio increases, the etch rate of the non‐exposed resist increases from 2 nm/min at 0% of DR (0 µsec) to 4.8 nm/min at 15% of DR (300 µsec). Similarly, the exposed resist shows an increase of ER from 0.4 nm/min at 0% of DR (0 µsec) to 1.6 nm/min at 15% of DR (300 µsec). As a result, the development selectivity decreased with increasing DR of the bias pulse from 5 to 3.0, as summarized in Figure 5c. This behavior highlights the increasing contribution of the physical sputtering effect under longer duration of bias DR, which enhances energetic ion‐induced etching in both non‐exposed resist and exposed resist.

The XPS analysis in Figure 5d shows that increasing the bias duty ratio (DR) leads to a decrease in both F and Cl contents on the film surfaces, indicating ion‐induced desorption of adsorbed species. For the non‐exposed films, the atomic percentage of F decreases from ∼6.0% at a bias DR of 0% to ∼4.3% at 15%. In contrast, the Cl content exhibits a non‐monotonic behavior: it increases slightly from ∼22.0% at 0% bias DR to a maximum of ∼24.9% at a bias DR of 2.5%, followed by a gradual decrease to ∼20.1% at 15%.

The initial increase in Cl atomic concentration at a bias DR of 2.5% can be attributed to the competing roles of ion bombardment and chemical removal. While the applied bias power promotes ion‐assisted removal of Sn in the non‐exposed regions, the relatively short bias DR limits the cumulative ion bombardment time, reducing the effectiveness of etch‐assisted desorption. As a result, Sn is partially chlorinated and remains on the surface in the form of non‐volatile or weakly volatile SnCl_x_ species (x < 4), leading to an elevated Cl atomic percentage detected by XPS. With further increases in bias DR, prolonged ion bombardment enhances etch assistance and increases the etch rate (ER), enabling more complete removal of Sn as volatile by‐products. Consequently, the surface concentration of residual Cl decreases. However, the increased ER at higher bias DR is also accompanied by a tendency toward reduced selectivity, reflecting the stronger physical sputtering component under extended ion bombardment.

In contrast, the exposed films display only modest compositional changes as a function of bias DR. The F atomic percentage slightly decreases from ∼3.6% at 0% to ∼3.1% at 2.5%, suggesting preferential removal during the initial application of bias. With further increases in bias DR, the F content remains nearly constant (∼3.3%), indicating that the surface chemistry reaches a quasi‐saturated state in which additional ion bombardment has minimal impact. The Cl content follows a similar trend, decreasing from ∼4.3% at 0% to ∼3.1% at 2.5% and remaining nearly unchanged at higher bias DR (∼3.3%). These observations suggest that, for exposed films, bias DR predominantly influences physical sputtering rather than chemical adsorption or reaction‐limited processes.

Narrow scan spectra (Figure 5e for F 1s and (f) for Cl 2p) were consistent with these findings: In non‐exposed films, C‐F related bindings (Figure 5e) and C‐Cl bindings (Figure 5f, organic‐Cl) were gradually decreased with higher bias DR. Both chemical compounds on the surface were relatively more volatile than metal compounds by ion bombardment. In the case of exposed films, only minor changes are shown. These results clearly demonstrate that the bias pulse mainly acts as the physical removal step of surface species through ion bombardment, eventually leading to a reduction in overall development selectivity at higher bias DRs. Also, Figure S9 shows the ratio of the organic‐Cl to metal‐Cl peak areas in Figure 5f of non‐exposed resist as a function of bias pulse DR. As the bias duty ratio increases, the relative contribution of organic‐Cl bonding decreases, indicating that energetic ion bombardment effectively removes weakly bonded carbon‐chlorine species from the surface.

Overall, Figures 4 and 5 demonstrate the independent controllability of radical and ion contributions in a pulsed plasma. By tuning the source pulse duty ratio, the chemical adsorption of reactive radicals can be effectively modulated to selectively etch the non‐exposed region. Meanwhile, the bias pulses control ion bombardment and must be minimized to preserve the selectivity. These findings are consistent with the previous research [34, 40, 41, 42], which has shown that pulsed plasma operation allows temporal separation of radical and ion fluxes through appropriate modulation of pulse parameters.

To evaluate the practical merits of this pulsed plasma development strategy, a direct comparison with conventional wet development is essential. Wet development, widely used for both CAR and metal oxide resist (MOR) platforms, typically offers high sensitivity and well‐defined contrast curves but suffers from critical issues such as capillary‐induced pattern collapse and environmental sensitivity. Therefore, in the following section, the performance of plasma and wet development methods is evaluated in terms of development contrast, CD control, and pattern fidelity.

**FIGURE 6:**
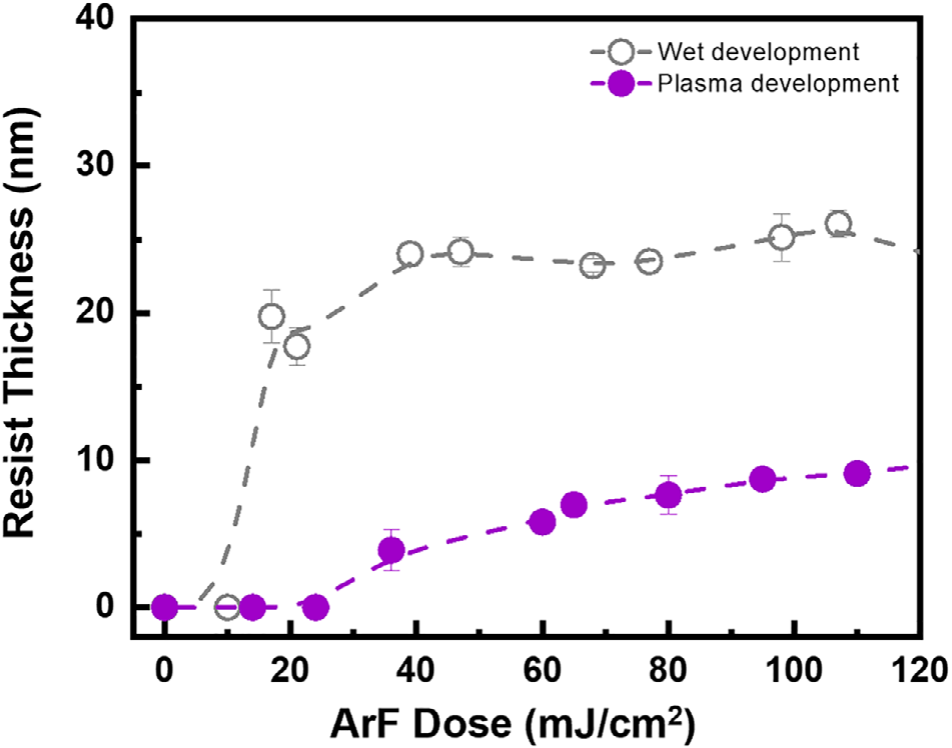
Contrast curve of dry resist exposed by ArF light source followed by development using wet development (gray empty circle) and pulsed plasma development (purple filled circle). Wet development was performed by dipping the exposed samples in a PGMEA + 2 wt.% formic acid solution for 5 sec. For pulsed plasma development, DR of the source pulse was set at 50% (1000 µsec) and DR of bias pulse at 2% (40 µsec) were used. V_max_ during the bias pulsing was set at ∼−75 V, and the samples were etched for 10 min.

Figure 6 presents the contrast curve of the microscale‐patterned dry resist exposed by the ArF source and developed either by wet development (gray open circles) or pulsed plasma development (purple filled circles). Wet development was conducted by dipping the samples in a solution composed of propylene glycol methyl ether acetate (PGMEA, 1‐methoxy‐2‐propanol acetate) + 2 wt.% formic acid for 5 sec, while the pulsed plasma process was conducted in a plasma formed with a 500 W source pulse DR of 50% (1000 µsec) and a −75 V_max_ bias pulse DR of 2% (40 µsec) for 10 min.

The wet‐developed resist exhibited a relatively high contrast, characterized by a sharp transition (at 17 mJ/cm^2^ of ArF DUV dose, ∼20 nm of film remains) and higher residual thickness (∼25 to 30 nm) across the entire dose range. In contrast, the plasma‐developed samples showed a gradual contrast slope and significantly reduced residual thickness (∼10 nm). At 24 mJ/cm^2^, the plasma process fully removed the resist, after which a gradual increase in remaining thickness was observed with increasing dose. These findings suggest that the lower selectivity of plasma development arises from partial removal of ambiguously reacted resist species via ion bombardment.

Figure 6 presents the contrast curve of the microscale‐patterned dry resist exposed by an ArF source and developed either by wet development (gray open circles) or pulsed plasma development (purple filled circles). Wet development was conducted by dipping the samples in a solution composed of propylene glycol methyl ether acetate (PGMEA, 1‐methoxy‐2‐propanol acetate) with 2 wt.% formic acid for 5 s, while the pulsed plasma process was performed using a plasma with a 500 W source pulse duty ratio (DR) of 50% (1000 µs) and a −75 V_max_ bias pulse DR of 2% (40 µs) for 10 min.

The wet‐developed resist exhibited a relatively high contrast, characterized by a sharp transition at an ArF DUV dose of 17 mJ/cm^−^^2^ (corresponding to ∼20 nm of remaining film thickness) and a higher residual thickness of ∼25–30 nm across the entire dose range. In contrast, the plasma‐developed samples showed a much more gradual contrast slope and a significantly reduced residual thickness of approximately 10 nm. At a dose of 24 mJ/cm^−^^2^, the plasma process completely removed the resist, after which a gradual increase in the remaining thickness was observed with further increases in dose. These results indicate that the lower selectivity of plasma development originates from partial removal of ambiguously reacted resist species through ion bombardment.

Plasma dry development inherently reduces selectivity relative to wet development due to ion‐bombardment‐induced physical etching, resulting in a thinner residual film after development. However, wet development suffers from an inherent limitation in its inability to effectively remove pattern bridging defects. Therefore, despite the reduced selectivity, plasma development is preferred for achieving high‐resolution, defect‐suppressed patterns.

**FIGURE 7:**
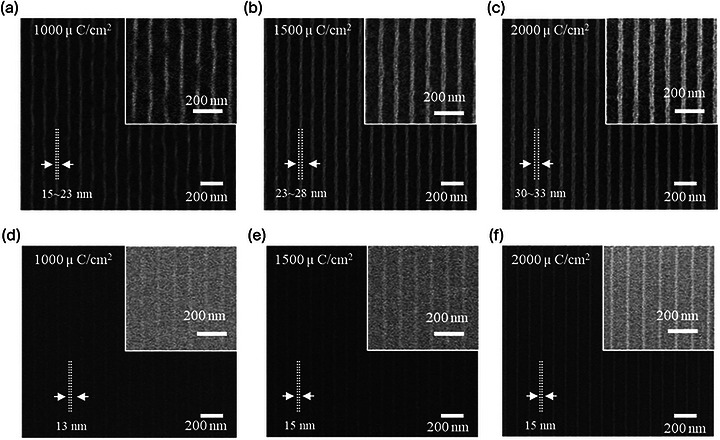
SEM images of e‐beam lithography line (10 nm) and space (50 nm) pattern after (a–c) wet development and (d–f) pulsed plasma development at various e‐beam doses. Samples were deposited by the CVD method on Si with a thickness of 38 nm. PEB was conducted at 250°C for 5 min on a hot plate. Wet development was performed by dipping the e‐beam exposed samples in a PGMEA + 2 wt.% formic acid solution for 5 sec. For pulsed plasma development of the e‐beam exposed samples, DR of source pulse set at 50% (1000 µsec) and DR of bias pulse at 2% (40 µsec) were used. V_max_ during the bias pulsing was set at ∼—75 V, and the samples were etched for 10 min.

Figure 7 shows SEM images of e‐beam lithography‐defined line (10 nm) and space (50 nm) patterns developed by using (a–c) wet development and (d–f) pulsed plasma development at various e‐beam doses ranging from 1000 to 2000 µC/cm^2^. At low doses (1000 µC/cm^2^, Figure 7a), wet development resulted in pronounced pattern wiggling. The measured line widths showed a broad distribution from 15 to 23 nm, indicating poor CD control and increased line width roughness. At the dose is increased to 1500 and 2000 µC/cm^2^ (Figure 7b,c), the line width expanded to ∼33 nm, which is attributed to enhanced energy transfer from the e‐beam [43, 44].

In contrast, the pulsed plasma‐developed patterns (Figure 7d–f) show significantly improved pattern fidelity across the entire dose range, especially in line width roughness and line wiggling. At 1000 µC/cm^2^ (Figure 7d), the line width is already well‐defined at ∼13 nm, and no residual scumming was observed, while contrast between space and pattern was small because of loss of pattern thickness by ion bombardment. Increasing the dose to 1500 and 2000 µC/cm^2^ (Figure 7e,f) also showed consistently straight line edges with stable CD (∼15 nm), demonstrating excellent dose‐to‐size and substantial reduction in LER, from 6.79 nm for wet development to 4.82 nm for pulsed plasma development at the same dose of 2000 µC/cm^2^. (The effects of pulse bias voltage V_max_ and duty % on the pulsed plasma development of the e‐beam exposed resist are shown in Figures S11–S13).

**FIGURE 8:**
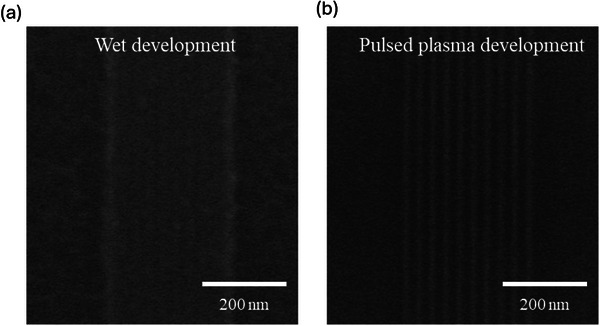
SEM images of e‐beam lithography line (10 nm) and space (30 nm) pattern after (a) wet development and (d) pulsed plasma development at 2000 µC/cm^2^ of e‐beam dose. Process conditions were the same as those in Figure 7.

Figure 8 compares SEM images of e‐beam lithography‐defined line (10 nm) and space (30 nm) patterns after (a) wet development and (b) pulsed plasma development at an e‐beam dose of 2000 µC/cm^2^. The process conditions for pulsed plasma development were identical to those used in Figure 7. As shown in Figure 8a, wet development failed to achieve clear pattern transfer at this narrow pitch, showing pronounced bridging and residual scumming. In contrast, even at such tight pitches, pulsed plasma development successfully transferred the 10 nm line/30 nm space patterns with clean space areas. The dry method's ability to reach into narrow spaces without capillary collapse is attributed to gas‐phase diffusion and directional ion bombardment.

**FIGURE 9:**
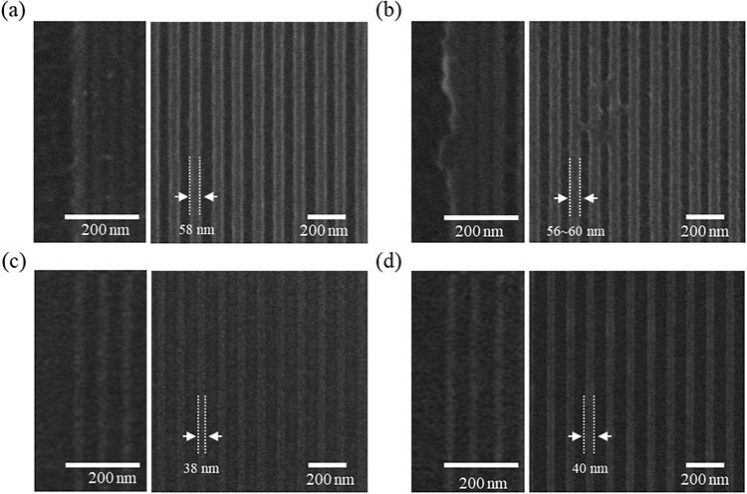
SEM images of line (50 nm) and space (50 nm) pattern. (a)∼(b) Wet development and (c)∼(d) pulsed plasma development at 1000 µC/cm^2^ of e‐beam dose. To investigate the effect of process delay on patterning performance, the samples ((b) and (d)) were stored in ambient air for 6 days (144 h) after the e‐beam lithography and PEB.

Also, to evaluate environmental tolerance and compare pattern stability after a delay in ambient air, samples were stored in ambient air for 6 days (144 h) after the e‐beam lithography and PEB performed at 250°C for 5 min on a hot plate in ambient air (Figure 9). As shown in Figure 9a, wet‐developed patterns showed CD variation, scumming, and roughness at isolated patterns after the delay. These results on the degradation of the patterning performance well agree with recent reports [32, 45, 46], which showed that a post‐exposure delay in ambient air induces H_2_O uptake, CO/CO_2_/O_2_‐driven Sn─O─Sn condensation, reducing the solubility of resist and causing CD variation in wet development. In contrast, plasma‐developed patterns showed a small increase in line CD (from 38 to 40 nm) after the same delay, maintaining patterning fidelity without scum and roughness of the line comparable to fresh samples. (As shown in Figure S14, the contrast curve of plasma‐developed resist with under‐exposure condition after 24 h showed an increase of sensitivity) This performance arises from the patterning behavior of the plasma process, in which radicals and ions easily remove AMC‐induced and PED‐related modifications of resist compared to wet development. Thus, pulsed plasma development offers intrinsic defect suppression capability, making it more suitable for high‐volume manufacturing where PED and environmental fluctuations must be tightly controlled.

**FIGURE 10:**
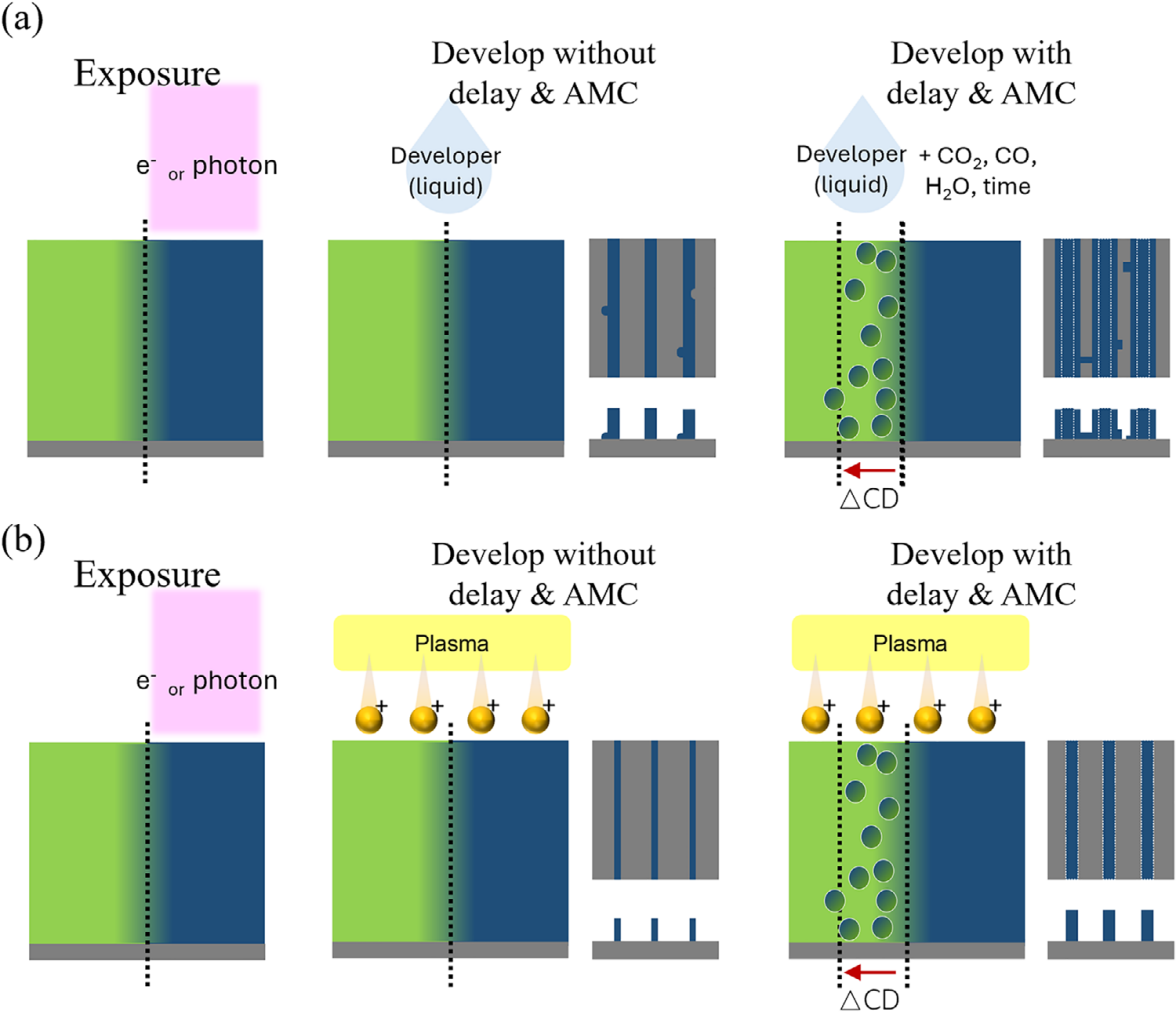
Schematic illustrations of development mechanisms of Sn‐based dry resist. (a) wet development and (b) plasma development using a pulsed plasma mode.

Based on the development results and surface analysis, the differences in the development mechanisms between wet development and pulsed plasma development for Sn‐based inorganic resist were schematically illustrated in Figure 10a,b, respectively. In both cases, the fundamental photochemical mechanism of metal oxide‐based resists originates from ligand cleavage induced by incident photons or electrons from lithographic sources [47, 48]. For example, in butyl‐tin cluster resists, increasing the dose leads to progressive cleavage of Sn–butyl bonds [49, 50], while in CVD‐deposited Sn‐alkyl materials, the Sn–alkyl substituents are similarly dissociated [26, 27].

In the case of wet development (Figure 10a), the mechanism relies on the solubility difference between exposed and unexposed regions. The unexposed area retains a C_x_Hᵧ‐rich environment, which remains soluble in developers, while the exposed area becomes enriched in metal–oxide bonds (e.g., Sn─O, Sn─OH), which are insoluble—thereby, resulting in a negative‐tone pattern. However, at narrow‐pitch structures, the developing solution creates harsh environments, often leading to pattern collapse and defects such as scumming and bridging. Moreover, process delay and AMCs further change the resist chemistry. These environmental effects accelerate detachment of ligands and the formation of oxidized species [30, 31, 32]. Although such modifications might seem to enhance resist sensitivity, they induce critical dimension (CD) variation and increase the probability of bridging and line‐edge roughness in nanoscale patterns.

In the case of pulsed plasma development (Figure 10b), its main development mechanism originates from the different formation rate of etch by‐products between non‐exposed and exposed regions. In other words, in the non‐exposed region, highly reactive radicals such as Cl, H, F, CF_x_, etc. from the plasma rapidly generate volatile by‐products (Sn─Cl_x_, Sn─H_x_, C─O, C─F, H─O, etc.) [51, 52], and these are easily removed from the surfaces. For the exposed regions, due to the higher binding energy of the metal oxo network, the removal rate of the exposed region is slower than that of the non‐exposed regions, resulting in negative tone development. (See Figures S15 and S16; spin‐coated Sn‐based resist could also be developed similarly by a pulsed plasma) In addition, directional ions and gas‐phase etchant (radicals) could easily get into the bottom of small‐pitch patterns and remove non‐exposed materials by forming gas‐phase by‐products. These are the reasons that plasma development could prevent pattern collapse and residues while minimizing the CD variation from external contaminations.

## 4 Conclusions

In this study, we demonstrated a pulsed plasma development process as an effective development strategy for Sn‐based inorganic resists. By decoupling the application of the source and bias RF powers during the pulsed plasma development, the process enabled selective removal of non‐exposed regions while preserving exposed resist, patterned by both ArF and e‐beam lithography. This resulted in clear negative‐tone behavior with minimal pattern distortion.

For the plasma development, unlike continuous wave (CW) plasma, which induces excessive ion bombardment and surface chemical mixing, the pulsed plasma process suppressed damage and enhanced development selectivity by its cyclic etching behavior. The control over pulse parameters revealed that increasing the source duty ratio enhanced the surface adsorption of reactive neutral species (e.g., Cl, H, CH, etc.). This selectively increased the etch rate of non‐exposed regions. In contrast, the increase of the duty ratio and power (or voltage) of the bias pulse had little effect on the chemical composition of both films. This is attributed to the low‐density capacitively coupled plasma (CCP)‐like nature of the plasma, and its dominant mechanism is physical sputtering rather than chemical reactions during the bias pulse period. As a result, compared to wet development, the line‑and‑space patterns developed using pulsed plasma development exhibited a reduction in LER from 6.79 to 4.82 nm, corresponding to a 29% improvement in LER.

Importantly, the vacuum‐based and liquid‐free environment of the plasma process inherently reduced the effects of AMC and resist aging after the lithography process, which are critical sources of defectivity such as residues and bridging between patterns in conventional wet development processes. In wet development, resist modification due to AMC and aging could induce ligand dissociation and the formation of Sn─O─X‐related species, resulting in exposed‐like regions, particularly at the boundaries between exposed and non‐exposed regions in small pitch patterns. These exposed‐like regions, which were not effectively removed by liquid developers—especially in narrow‐pitch structures—leading to defect formation (bridging, scum, etc.). Plasma development, by contrast, uses reactive ions capable of penetrating dense features and removing AMC‐induced residues, thereby preserving pattern fidelity and minimizing defects.

Moreover, the proposed strategy is universally applicable to both vapor‐deposited dry resists (e.g., via CVD, atomic layer deposition (ALD), or molecular layer deposition (MLD)) and spin‐coated metal oxide resists, regardless of film density or structure. The decoupled plasma exposure sequence—alternating between radical chemisorption and directional ion bombardment—offers enhanced process control and selectivity. Although the experimental demonstrations were performed using DUV and electron‐beam exposures, the underlying resist reaction mechanisms are fundamentally equivalent to those in EUV lithography, suggesting that this approach is directly applicable to EUV‐patterned photoresists [53]. Overall, the pulsed plasma development extends the process window while enhancing both reliability, scalability, and defect tolerance. These findings originate from the cyclic behavior of the pulsed plasma, and could be utilized as a highly selective, low‐damage, and defect‐suppression patterning solution for next‐generation lithography platforms.

## Conflicts of Interest

The authors declare no competing financial interests.

## Supporting information




**Supporting File**: smtd70794‐sup‐0001‐SuppMat.docx.

## Data Availability

Research data are not shared.
