# Supplementary material for: Dry Development of Dry Coated Sn‐Based Inorganic Resist for Defect‐Suppressed and High‐Resolution Patterning Process
Source: Small Methods. 2026 Jun 23;10(14):e70794. doi: 10.1002/smtd.70794 (PMC13397150; doi:10.1002/smtd.70794)
Supplement: Supplementary file 1 — Supporting File: smtd70794‐sup‐0001‐SuppMat.docx. [file SMTD-10-e70794-s001.docx]

**Supporting Information**

**Dry Development of Dry Coated Sn-based Inorganic Resist for Defect-suppressed and High-resolution Patterning Process**

Hee Ju Kim^1,†^, Min Cheol Kim^2,†^, Geun Young Yeom^1,3,*^

^1^ School of Advanced Materials Science and Engineering, Sungkyunkwan University, Suwon 16419, Korea

^2^ Department of Display Engineering, Sungkyunkwan University, Suwon 16419, Korea

^3^ SKKU Advanced Institute of Nano Technology (SAINT), Sungkyunkwan University, Suwon 16419, Korea

KEYWORDS.

Dry resist, Inorganic photoresist, Metal oxide resist, Pulsed plasma, Dry development, plasma development


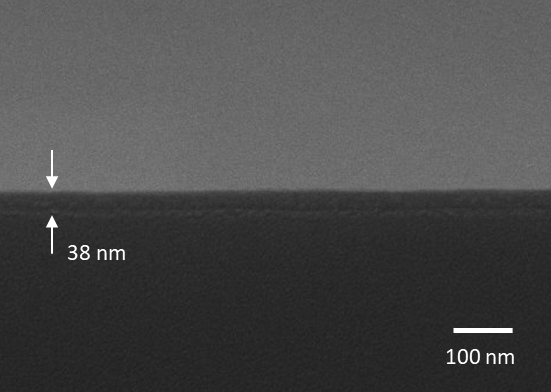


**Figure S1.** SEM image of Sn-based dry resist exposed by ArF scanner with the dose of 50 mJ/cm^2^

Figure S1 shows the thickness of Sn-based dry resist observed by cross-sectional SEM after exposed by an ArF scanner with the dose of 50 mJ/cm^2^.


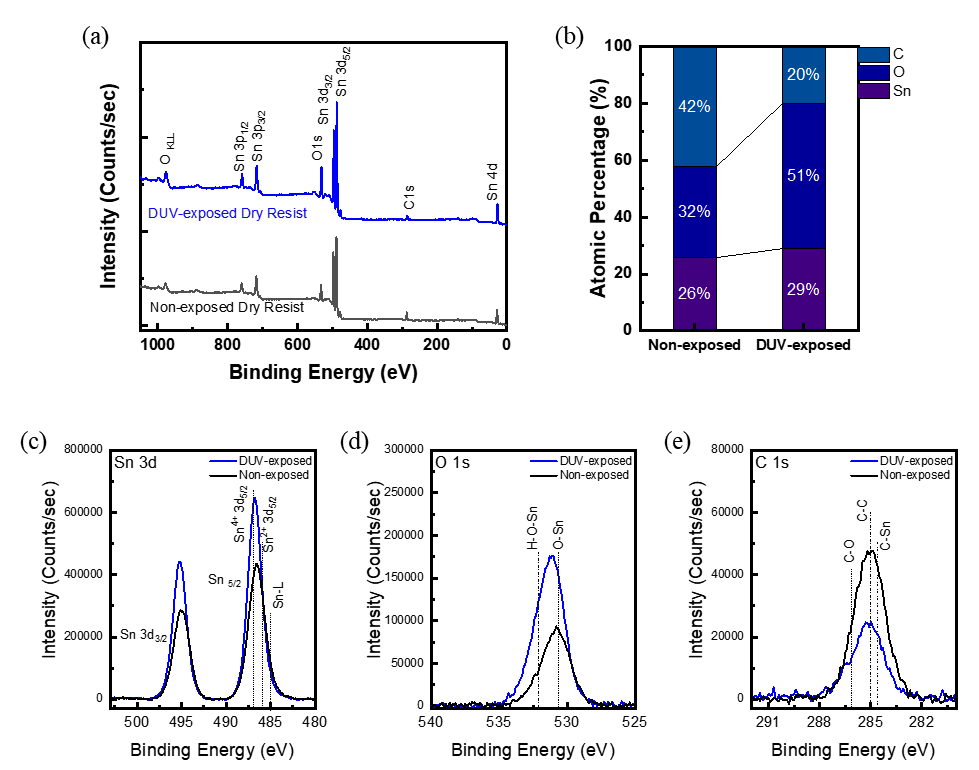


**Figure S2** (a) XPS wide scan spectra of DUV-exposed resist (blue) and non-exposed resist (black). (b) Atomic percentages of Sn, O, and C extracted from the wide scan XPS data, comparing DUV-exposed and non-exposed films. (c-e) narrow scan spectra of (c) Sn 3d, (d) O 1s, and (e) C 1s samples deposited by the CVD method on Si with a thickness of 38 nm. PEB was conducted at 250 ^o^C for 5 min on a hot plate. In the case of DUV-exposed resist, samples were exposed by DUV light without pattern.

Figure S2 (a) shows the wide scan spectra of DUV-exposed (blue) and non-exposed (black) resists. In both cases, major elements were detected include Sn, O, and C. Especially, intensity of Sn and O peaks increases after the DUV exposure, while C peak decreases, suggesting carbon ligand cleavage and condensation by light exposure and baking. This trend is quantitatively supported by the atomic percentage data in Figure S2 (b). After the DUV-exposure and PEB, the wt.% of O increased from 32% to 51% and the at % of C decreased from 42 % to 20 %. These results indicate that the DUV exposure promotes ligand cleavage and removal of the organic moieties in the resist, resulting in a more metal-oxide rich structure.

Narrow scan spectra of (c) Sn 3d, (d) O 1s, and (e) C 1s also support the analysis results. As shown in Figure S2 (c), after the light exposure and bake, increased peak intensity and peak shifts of Sn 3d could be observed, resulting from the change to the Sn oxidation state^1,2^. Figure S2 (d) shows the increase in O 1s signal^2^, particularly Sn-O-H binding region, which is the result of condensation reaction with H_2_O, O_2_ in ambient air. In contrast, C 1s peak^2^, shown in Figure S2 (e), decreases after the light exposure and baking, suggesting the loss of organic ligands and cross linking due to photon-induced or thermal-induced desorption.


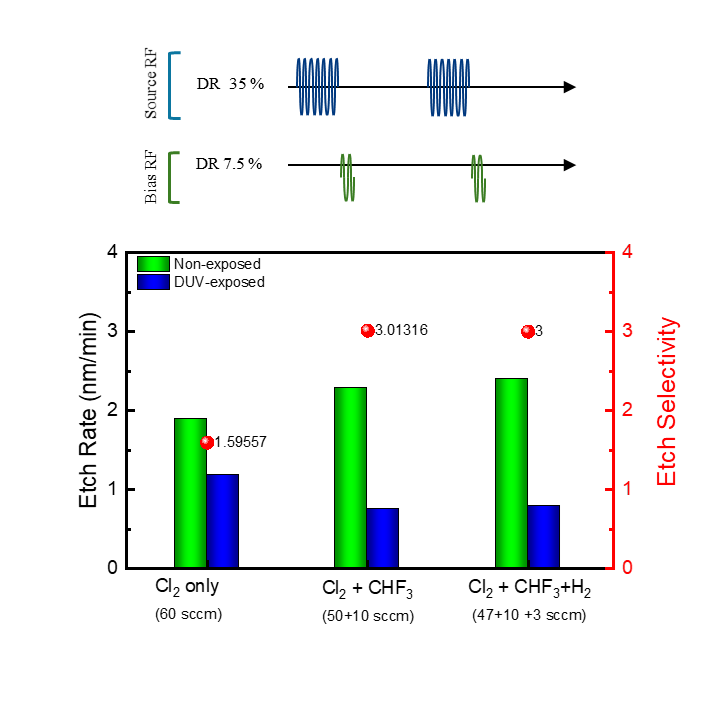


**Figure S3.** Etch rate and etch selectivity of non-exposed and DUV-exposed resists under different gas chemistries using a pulsed plasma. All other process conditions were the same as those in Figure 3. Four gas combinations were tested: Cl₂ only (60 sccm), Cl₂ + CHF₃ (50 + 10 sccm), and Cl₂ + CHF₃ + H₂ (47 + 10 + 3 sccm).

To explain the use of Cl₂ + CHF₃ + H₂ gas mixture in all pulsed plasma development experiments in the main manuscript, a comparative analysis of etch rate and development selectivity was conducted for four different gas chemistries. As shown in Figure S3, while Cl₂ alone provides moderate etch rate and low selectivity (1.60), the addition of CHF_3_ improves ER of non-exposed resist and decreases ER of exposed resist, resulting in increase of selectivity. This results indicate that the presence of CHF_x_, CF_x_, and related species in the plasma promotes the removal of non-exposed resist, which consists of carbon ligands and weakly bonded metal-oxygen moieties, resulting in the formation of volatile by-products. Simultaneously, these species suppress the reaction of the exposed resist by forming polymeric fluorocarbon layers on its surface. To further enhance the removal of the non-exposed region while preserving development selectivity, a small amount of H₂ was added. As a result, the etch rate of the non-exposed resist increased from 2.29 nm/min to 2.40 nm/min, while that of the exposed resist slightly increased from 0.76 nm/min to 0.80 nm/min.


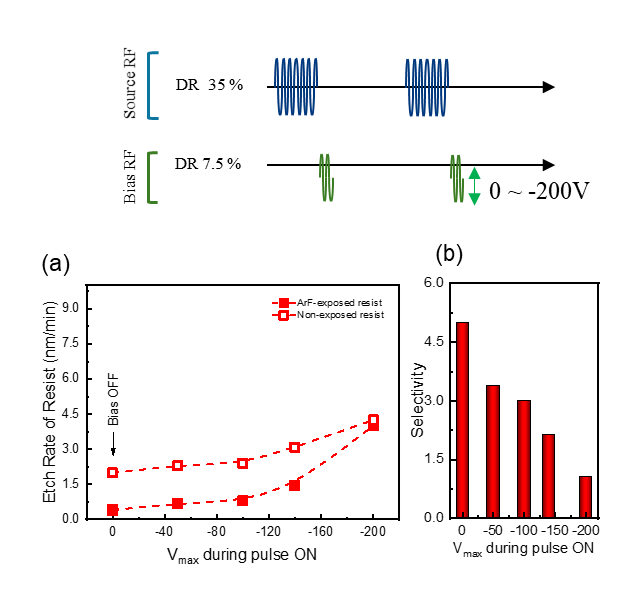


**Figure S4** Effect of bias voltage on (a) the etch rates of non-exposed and exposed resists and (b) development selectivity. All other process conditions were the same as those in Figure 3.

Figure S4 shows the etch rates of the non-exposed resist and exposed resist. Etch rates of both films increased with higher bias voltage. However, the development selectivity decreased with increasing bias voltage from 5 to 1.1.


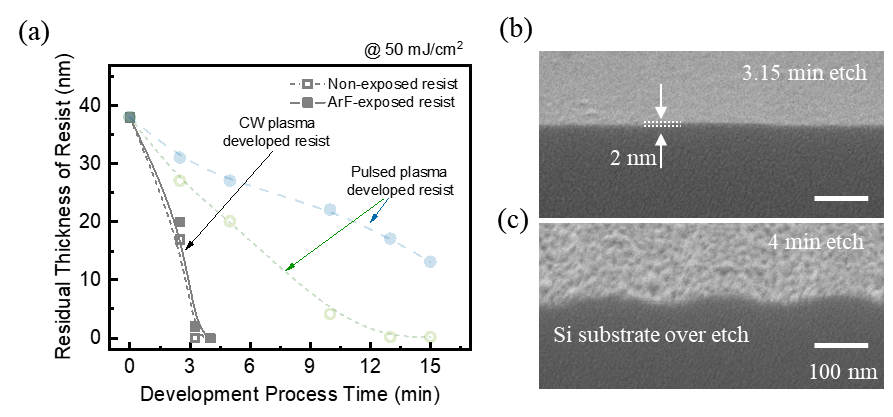


**Figure S5** (a) Thickness change of 500 nm line patterns as a function of plasma development process time under a continuous wave (CW) plasma, compared with the pulsed plasma development (data from Figure 3). (b) and (c) Cross-sectional SEM images of line pattern after 3.15 min and 4 min of CW plasma development, respectively. (CW plasma process conditions: RF power 500 W, bias voltage -120 V_max,_ Cl_2_ 47 sccm, CHF_3_ 10 sccm, H_2_ 3 sccm, and operating pressure 100 mTorr)

As shown in Figure S5, the CW plasma development resulted in a significantly faster etch rate for both the non-exposed and ArF-exposed resist compared to the pulsed plasma. After 3.15 min etching, only ~2 nm of resist remained in the exposed resist, and further etching led to substrate over etching of Si substrate, as in the SEM image of Figure S5 (c). This highlights the importance of decoupled plasma control offered by the pulsed plasma for preserving the exposed resist pattern and minimizing etching of underlayer.


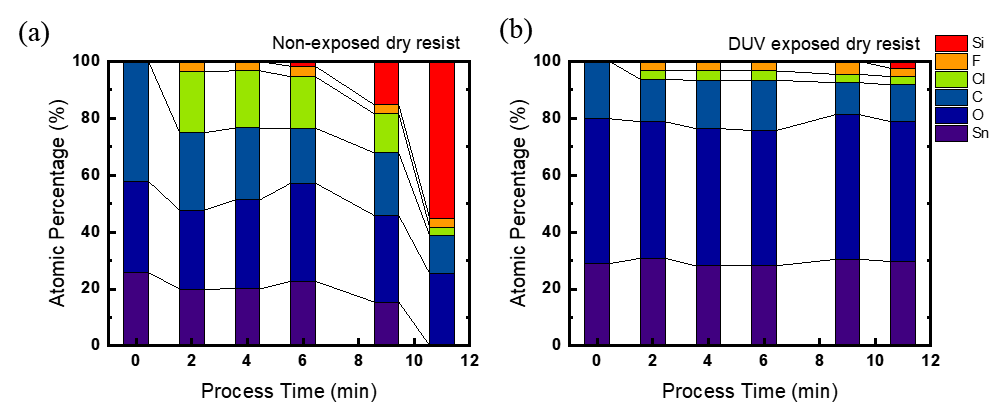


**Figure S6.** Change in atomic percentage for (a) non-exposed resist and (b) DUV-exposed resist observed by XPS as a function of pulsed plasma development time

Figure S6 shows the change in atomic percentage for (a) non-exposed resist and (b) DUV-exposed resist observed as a function of pulsed plasma development time for the conditions in Figure 3.


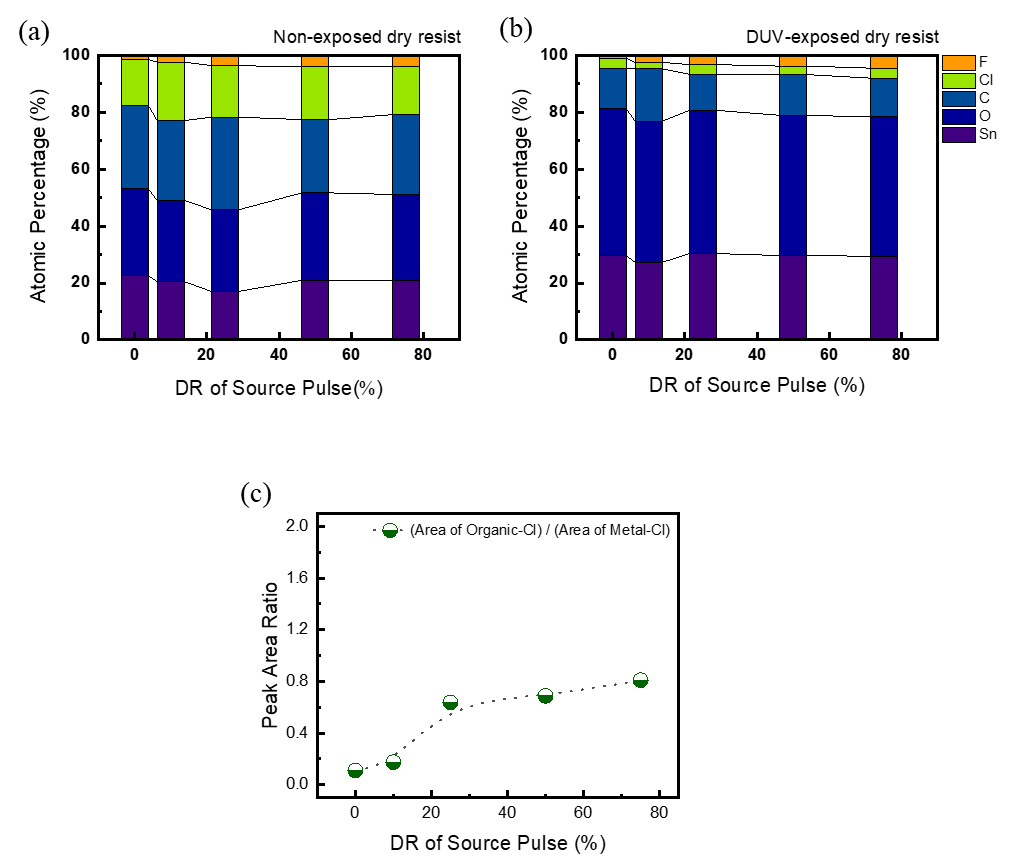


**Figure S7** Change in atomic percentage as a function of source pulse duty ratio from Figure 4; (a) non-exposed resist and (b) DUV-exposed resist. (c) Peak area ratio at Cl 2p of non-exposed resist (area of organic-Cl/area of metal-Cl)

Figure S7 (a) and (b) show the atomic percentage change of non-exposed resist and exposed resist as a function of source pulse DR. Figure S7 (c) presents the peak area ratio of organic-Cl (C-Cl) to metal-Cl (Sn-Cl) in the Cl 2p narrow scan spectra for non-exposed resist in Figure 4(f). As the source DR increases, the relative contribution of organic-Cl bonding increases, suggesting enhanced reaction between carbon-containing ligands in the resist and plasma-generated Cl species.


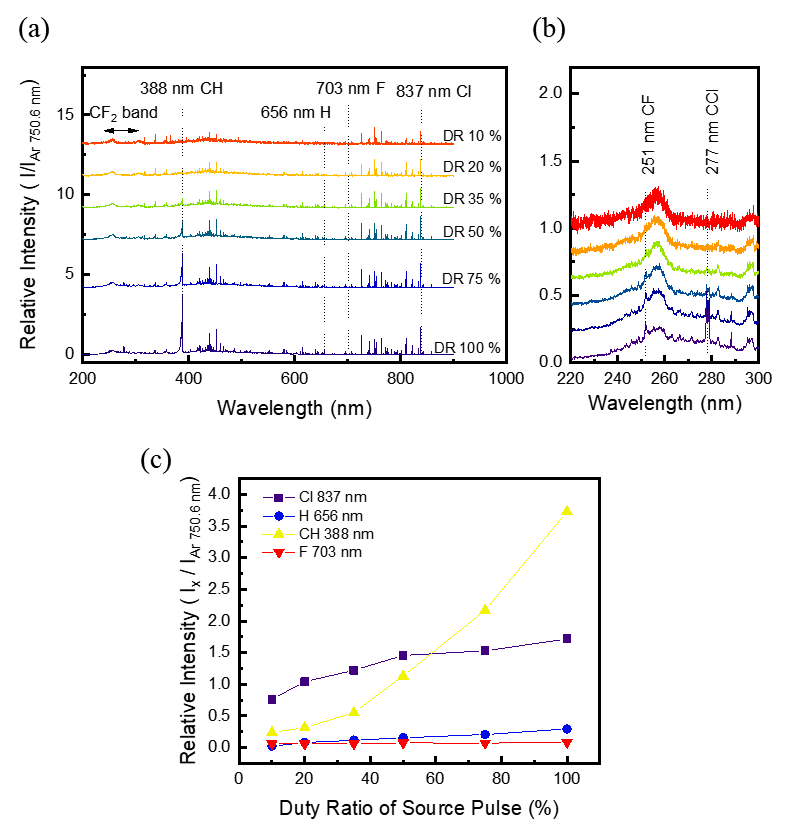


**Figure S8**  Normalized OES spectra for various DRs of source pulse when the bias pulse DR was kept at 7.5 %. (b) OES spectra from 220 nm to 300 nm (CF_x_ band, CF: 251 nm, and CCl 277 nm), and (c) relative intensities of Cl (837 nm), H (656 nm), CH (388 nm), and F (703 nm) peaks as a function of DR of source pulse^3–7^

Figure S8 shows the optical emission spectroscopy (OES) results for various source pulse duty ratios to analyze the generation of reactive species in the plasma. OES spectra measured from 200 to 900 nm reveal intensity variations of key plasma species including CH (388 nm), H (656 nm), F (703 nm), and Cl (837 nm), showing an increase in emission intensity as the duty ratio of the source pulse increases. CF (251 nm) and CCl (277 nm) emissions were also observed in Figure S8 (b), which are important indicators of fluorocarbon and chlorocarbon species generation. Quantitative trend analysis shows that the relative intensities of Cl, H, and CH species increase with higher duty ratios, suggesting enhanced radical generation under longer plasma ignition times. These results support the XPS findings in Figure 4, indicating that reactive radical flux is strongly modulated by the source pulse duty ratio.


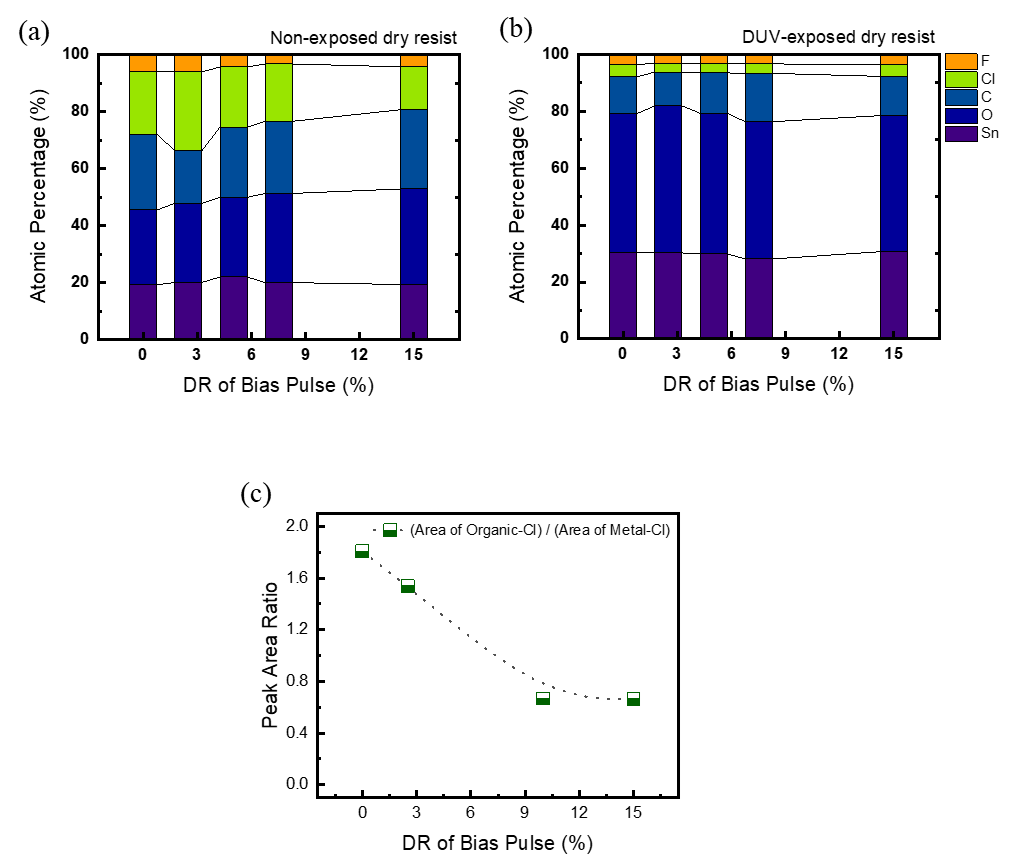


**Figure S9** Change in atomic percentage as a function of bias pulse duty ratio from Figure 5; (a) non-exposed resist and (b) DUV-exposed resist. (c) Peak area ratio at Cl 2p of non-exposed resist (area of organic-Cl/area of metal-Cl)

Figure S9 (a) and (b) show the atomic percentage change of non-exposed resist and exposed resist as a function of source pulse DR. Figure S9 (c) presents the peak area ratio of organic-Cl (C-Cl) to metal-Cl (Sn-Cl) in the Cl 2p narrow scan spectra for non-exposed resist in Figure 5(f). As the bias duty ratio increases, the relative contribution of organic-Cl bonding decreases, indicating that energetic ion bombardment effectively removes weakly bonded carbon-chlorine species from the surface.


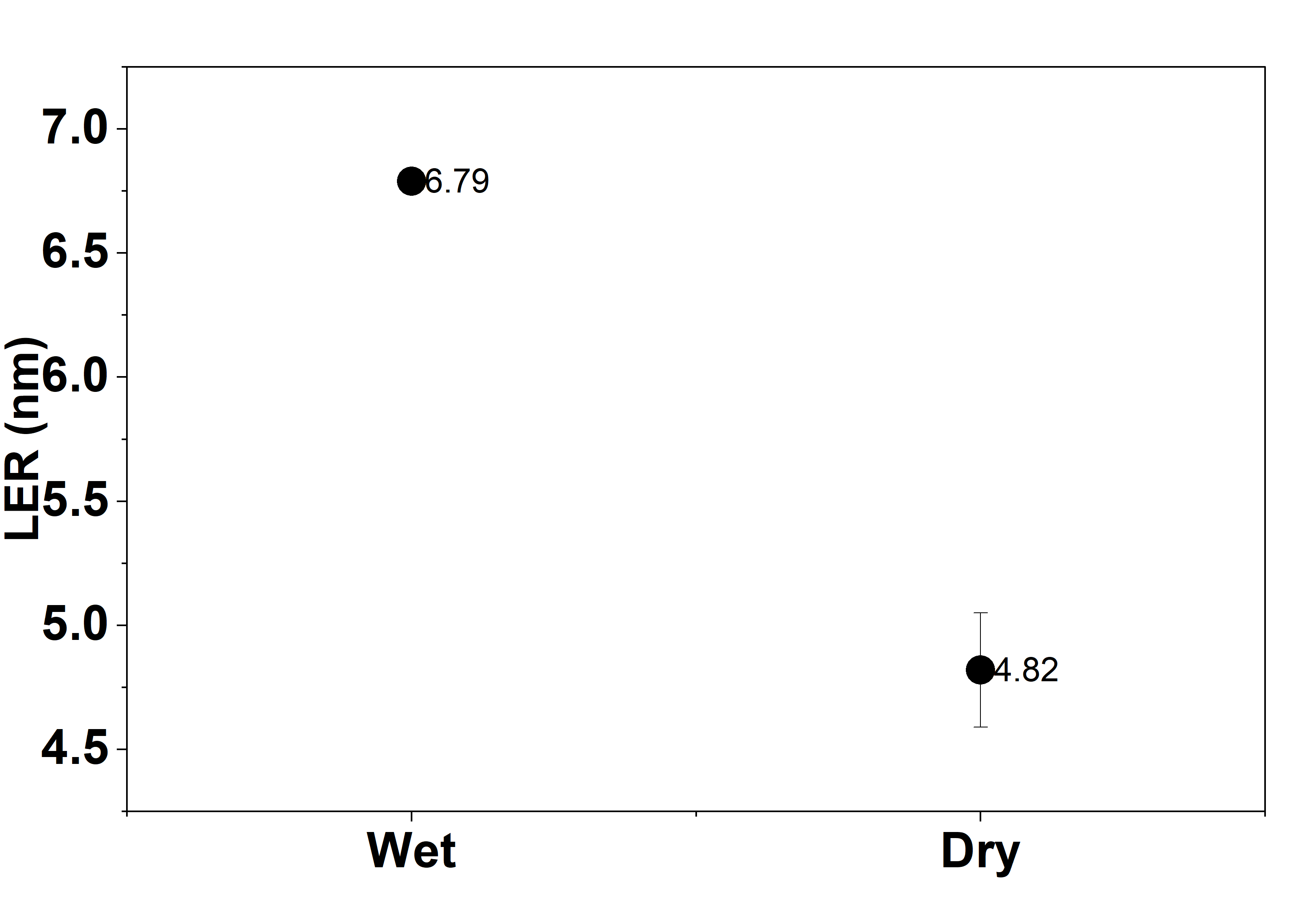


**Figure S10.** Comparison of LER for line‑and‑space patterns developed by wet development and pulsed plasma development at a dose of 2000 μC/cm². Wet development was performed by dipping the e-beam exposed samples in a PGMEA + 2 wt % of formic acid solution for 5 sec. For pulsed plasma development of the e-beam exposed samples, DR of source pulse set at 50 % (1000 $\mu$sec) and DR of bias pulse at 2 % (40 $\mu$sec) were used. V_max_ during the bias pulsing was set at ~ - 75 V, and the samples were etched for 10 min.

Figure S10 shows a comparison of the LER of patterns developed using wet development and pulsed plasma development. The LER was calculated using the Lacerm software. At an e‑beam dose of 2000 μC/cm², the pattern developed using a wet solution exhibited an LER of 6.79 nm, whereas the pattern developed by dry (pulsed plasma) development showed a reduced LER of 4.82 nm.


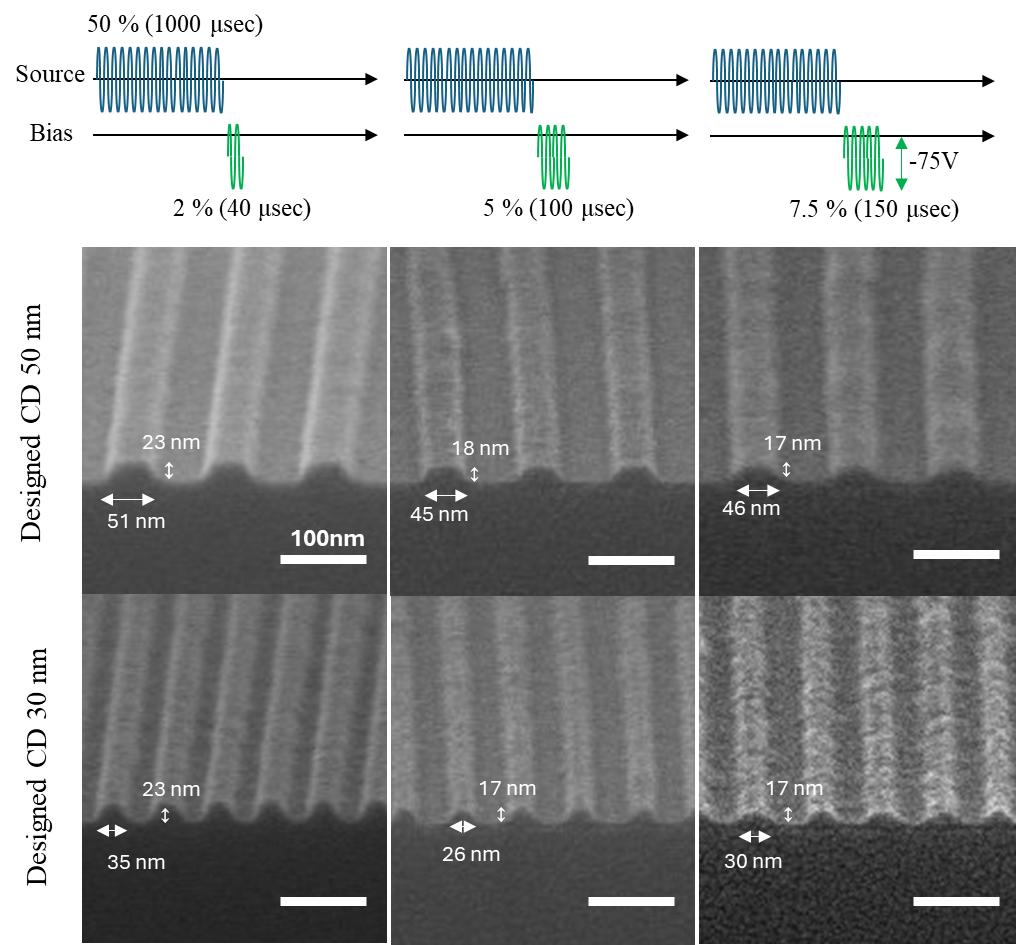


**Figure S11** Tilted cross-sectional SEM images of dry resist after the plasma development processes at different bias pulse DRs. Samples were exposed by an e-beam source with the dose of 2000 μC/cm^2^. PEB was conducted at 250 ^o^C for 5 min on a hot plate. During the process, the source pulse was set to 1000 µsec with PRF of 500 Hz (DR: 50 %) and V_max_ during the bias pulse was approximately kept at -75 V.

Figure S11 shows the effect of bias pulse duty ratio on the exposed PR edge profile. As the DR of bias pulse increases, reactive ions flux increase, causing PR edge rounding and lower development selectivity.


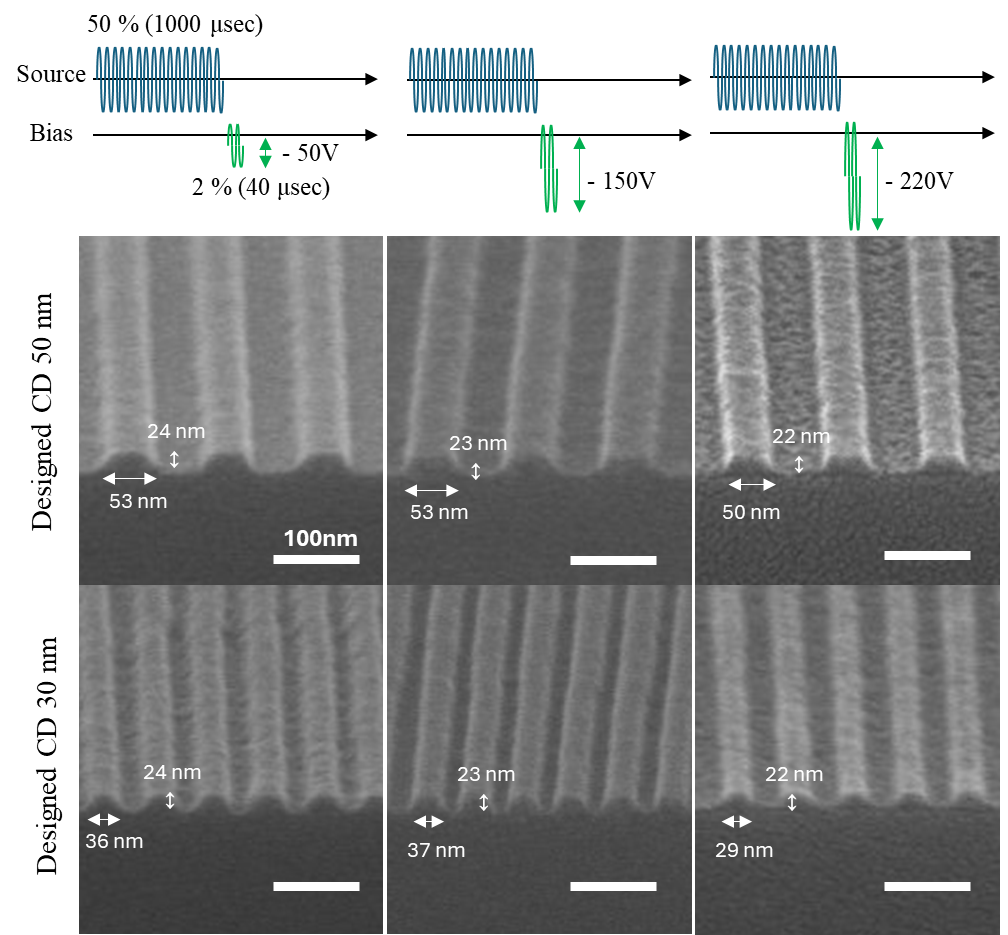


**Figure S12** Tilted cross-sectional SEM images of dry resist after plasma development processes at different bias pulse V_max_. Samples were exposed by an e-beam source with the dose of 2000 μC/cm^2^. PEB was conducted at 250 ^o^C for 5 min on a hot plate. During the process, the source and bias pulse were set to 1000 µsec and 40 µsec with PRF of 500 Hz (DR: 50 % and 2 %, respectively).

Figure S12 shows tilted cross-sectional SEM images of e-beam patterned resists after plasma development at different V_max_ (−50 V, −150 V, and −220 V) under fixed pulse conditions (source: 50%, 1000 μsec; bias: 2%, 40 μsec). As the bias voltage increases, the thickness of the line pattern slightly decreases, indicating the increased ion-induced physical erosion. However, reducing the bias voltage to ~0 V_max_ to prevent erosion of the pattern edge may cause footing or residue remaining on the developed area of the resist (as discussed in Figure S12).

**
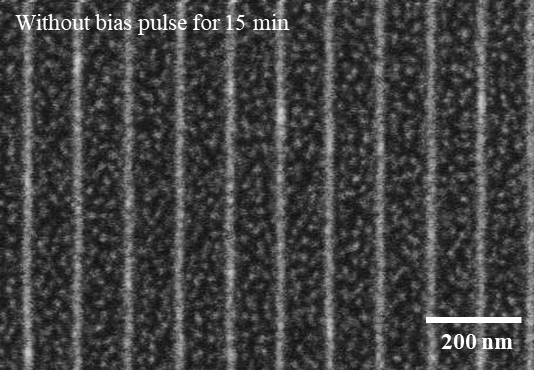
**

**Figure S13.** SEM image of plasma development without bias pulse

Figure S13 shows the SEM image after plasma development without bias pulse for 15 min. Samples were patterned with the same conditions as in Figure 7 (f) (2000 µC/cm^2^). Compared to Figure 7(d) ~(f), Figure S13 clearly shows increased surface roughness at the bottom of the patterns, which is likely caused by residuals remaining due to the absence of bias pulse during plasma development despite the highest selectivity (5, shown in Figure S4 (b)).

From the development results of Figures S11 to S13, to increase the development selectivity, ion bombardment must be minimized, but an appropriate amount of biasing is required to properly remove the residue. Given this trade-off, the experiments of Figures 6 ~ 9 were conducted using the scheme of source DR 50% and bias DR 2% to obtain optimal results.


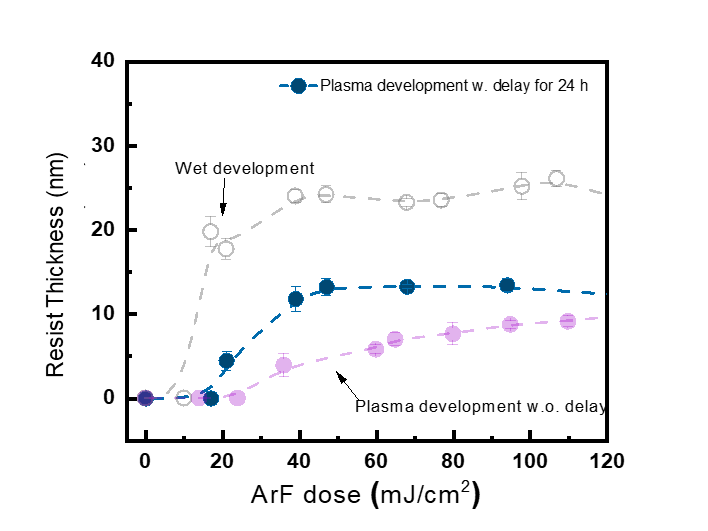


**Figure S14** Contrast curve of plasma developed ArF DUV-exposed patterns. Samples were stored in ambient air for 24 hour after ArF lithography and PEB process. Gray empty circles and purple filled circles are the samples developed by wet and pulsed plasmas, respectively, without any delay in Figure 6.

Figure S14 shows the contrast curve of ArF-exposed resist developed by pulsed plasma after a 24-hour post-exposure delay in ambient air. Compared to the plasma-developed sample without delay (purple filled circles, Figure 6), the delayed sample (dark blue filled circles) exhibited higher residual thickness across all dose ranges, indicating that ambient aging increases the reaction of carbon ligand with CO_2_, H_2_O and CO^8^. As a result, these modified areas appear to act like the area exposed to a higher dose. So, AMC and post-exposure delay make it appear as if the sensitivity has been increased without an additional exposure to a light source. But still, due to the ion bombardment, the chemical contrast was lower than that of the wet developed result.


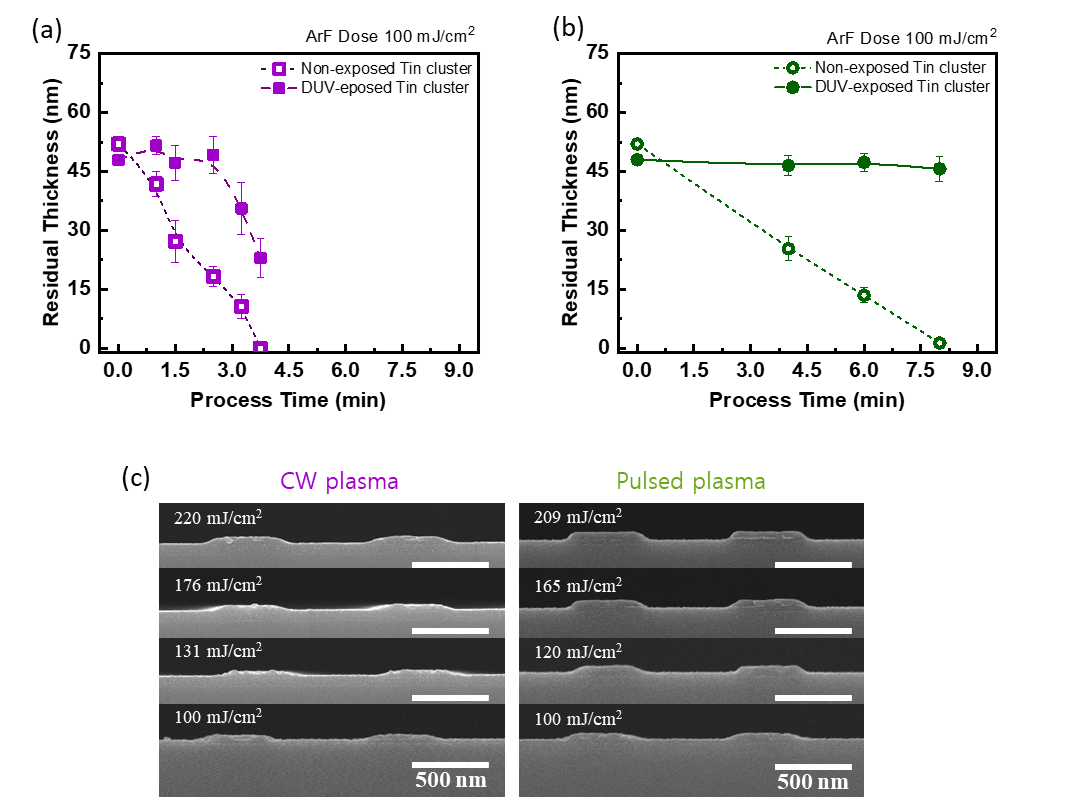


**Figure S15.** Thickness changes of spin-coated [(BuSn)_12_O_14_(OH)_6_](OH)_2_ (Sn OH) as a function of development process time using (a) a CW plasma and (b) a pulsed plasma method. (c) Sidewall SEM images of 500 nm line patterns of [(BuSn)_12_O_14_(OH)_6_](OH)_2_ with various ArF exposure doses.

In addition to Sn-based dry resist, to investigate the development characteristics of spin-coated Sn OH resist films during the process with (a) a CW plasma and (b) a pulsed plasma, thickness changes of non-exposed and exposed Sn OH were measured by SEM and are shown in Figure S15 as a function of process time.

During the CW plasma development process, instant RF power of the source was kept at 250 W, and V_max_ during the pulse period was fixed at - 140 V. A gas mixture of Cl_2_/CF_4_ (60/25 sccm) at 45 mTorr was used and the substrate temperature was kept at 0 ^o^C. In case of the pulsed plasma conditions, all of conditions were the same as the CW conditions, except the pulse condition. The source pulse was kept at 700 µsec (PRF 1 kHz, DR 70 %) and the bias pulse was applied at 150 µsec DR 15 %). at the end of the source pulse. For this experiment, the resist film was coated at 2000 rpm for 20 sec on 8 inch diameter silicon wafer. Soft bake was conducted at 90 ^o^C for 3 min. After that, the films were exposed to ArF light source with the dose of 100 mJ/cm^2^ and post exposure bake was conducted at 90 ^o^C for 3 min.

As shown in Figure S15 (a), at the CW plasma condition, the thicknesses of non-exposed (empty square) and DUV-exposed (filled square) Sn OH were gradually decreased as the process time increases. ERs of non-exposed and exposed Sn OH were13.53 nm/min and 8.37 nm/min, respectively. Therefore, the development selectivity over non-exposed Sn OH was 1.62. In case of the pulsed plasma condition, as shown in Figure S15 (b), ERs of non-exposed and exposed Sn OH were slower than the CW plasma condition for both films by showing 6.03 nm/min and 0.14 nm/min, respectively, but the development selectivity was much higher as 41.99.

Figure S15 (c) shows the cross-section SEM images of 500 nm line developed patterns of Sn OH films for various DUV doses. Samples were exposed to ArF scanner with different doses from 100 mJ/cm^2^ to 220 mJ/cm^2^. Plasm development process conditions were the same as those in Figure S15 (a) and (b) except for development time. In case of CW mode condition, samples were developed in the CW plasma for 4 min, and for asynchronously pulsed plasma condition, patterned samples were developed for 8.25 min.

**
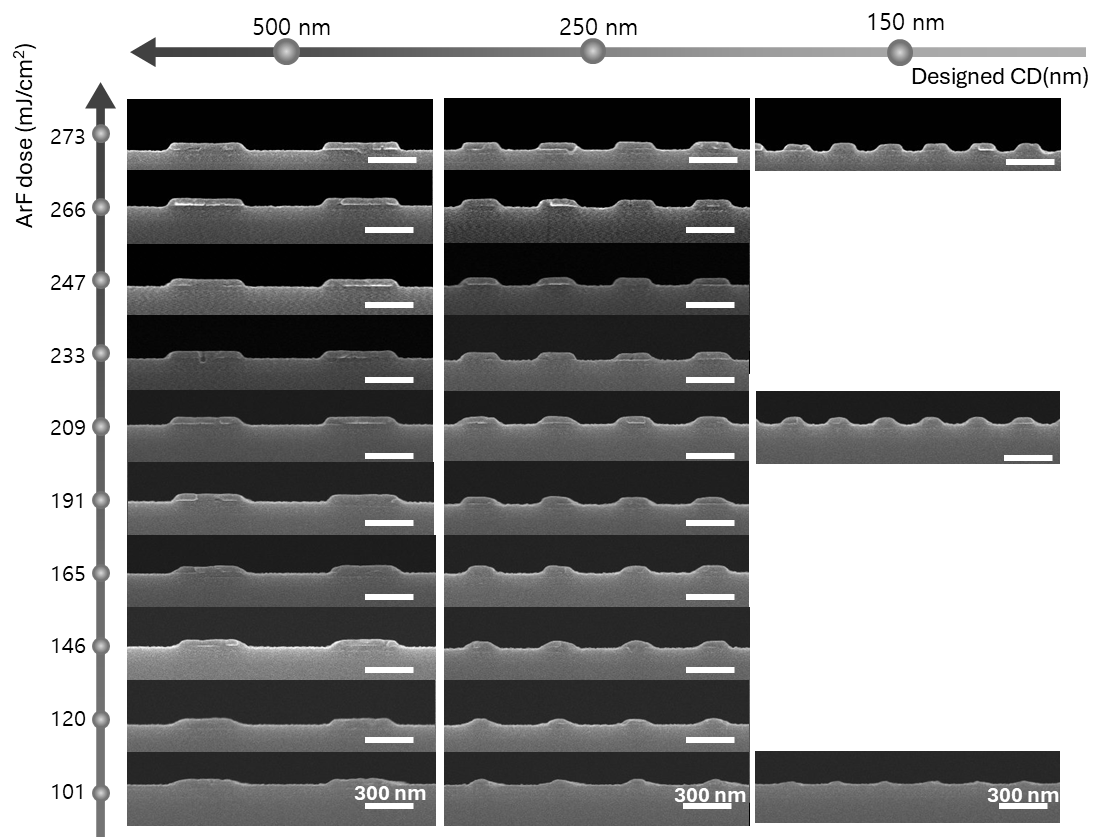
**

**Figure S16.** Cross-sectional SEM images of line and space patterns of ArF DUV-exposed [(BuSn)_12_O_14_(OH)_6_](OH)_2_ after pulsed plasma development with various ArF doses

Figure S16 shows the sidewall profiles of spin-coated Sn OH resist for different CDs exposed by ArF light source with various doses (101 mJ/cm^2^~273 mJ/cm^2^). Samples were treated with the same pulsed plasma conditions as in Figure S14 (b) for 8 min.

(1) Kwoka, M.; Ottaviano, L.; Passacantando, M.; Santucci, S.; Czempik, G.; Szuber, J. XPS Study of the Surface Chemistry of L-CVD SnO2 Thin Films after Oxidation. In *Thin Solid Films*; 2005; Vol. 490, pp 36–42. https://doi.org/10.1016/j.tsf.2005.04.014.

(2) Zhang, Y.; Haitjema, J.; Liu, X.; Johansson, F.; Lindblad, A.; Castellanos, S.; Ottosson, N.; Brouwer, A. M. Photochemical Conversion of Tin-Oxo Cage Compounds Studied Using Hard x-Ray Photoelectron Spectroscopy. *Journal of Micro/Nanolithography, MEMS, and MOEMS* **2017**, *16* (2), 023510. https://doi.org/10.1117/1.jmm.16.2.023510.

(3) Clarke, P. E.; Field, D.; Klemperer, D. F. Optical Spectroscopic Study of Mechanisms in CCl4 Plasma Etching of Si. *J Appl Phys* **1990**, *67* (3), 1525–1534. https://doi.org/10.1063/1.345663.

(4) Curtis, B. J.; Brunner, H. J. *End Point Determination of Aluminum CCI, Plasma Etching by Optical Emission Spectroscopy*.

(5) Alves, C.; Galvão, N. K. M.; Gregory, A.; Henrion, G.; Belmonte, T. OES during Reforming of Methane by Microwave Plasma at Atmospheric Pressure. *J Anal At Spectrom* **2009**, *24* (10), 1459–1461. https://doi.org/10.1039/b905323a.

(6) Zhang, H. Y.; Jin, C. G.; Yang, Y.; Ye, C.; Zhuge, L. J.; Wu, X. M. Structural and Electrical Properties of High-k HfO2 Films Modified by CHF3 and C4F8/O2 Plasmas. *Appl Phys A Mater Sci Process* **2014**, *117* (4), 2057–2065. https://doi.org/10.1007/s00339-014-8619-5.

(7) D’agostino, R.; Cramarossa, F.; De Benedictis, S.; Fracassi, F. *Optical Emission Spectroscopy and Actinometry in CC14-C12 Radiofrequency Discharges*; 1984; Vol. 4.

(8) Kenane, N.; Keszler, D. A. High-Resolution Lithographic Patterning with Organotin Films: Role of CO2in Differential Dissolution Rates. *ACS Appl Mater Interfaces* **2021**, *13* (16), 18974–18983. https://doi.org/10.1021/acsami.0c21942.
